# Cross-habitat interactions drive methylmercury contamination in a disturbed river ecosystem: novel metagenomic and biogeochemical insights

**DOI:** 10.1093/ismeco/ycag176

**Published:** 2026-06-23

**Authors:** Veronika Storck, Dominic E Ponton, Charlène Lawruk-Desjardins, Lise Millera Ferriz, Maxime Leclerc, Susanne Kraemer, Dolorès Planas, Marc Amyot, David Walsh

**Affiliations:** Biology Department, Concordia University, Montreal, QC H4B 1R6, Canada; Département de Sciences Biologiques, Université de Montréal, Montreal, QC H2V 0B3, Canada; Département de Sciences Biologiques, Université de Montréal, Montreal, QC H2V 0B3, Canada; Biology Department, Concordia University, Montreal, QC H4B 1R6, Canada; Département de Sciences Biologiques, Université de Montréal, Montreal, QC H2V 0B3, Canada; Département de Sciences Biologiques, Université de Montréal, Montreal, QC H2V 0B3, Canada; Biology Department, Concordia University, Montreal, QC H4B 1R6, Canada; Aquatic Contaminants Research Division, Environment and Climate Change Canada, Montreal, QC H2Y 2E7, Canada; Département de Sciences Biologiques, Université de Québec à Montréal, Montreal, QC H3C 3P8, Canada; Département de Sciences Biologiques, Université de Montréal, Montreal, QC H2V 0B3, Canada; Biology Department, Concordia University, Montreal, QC H4B 1R6, Canada

**Keywords:** sediment, periphyton, mercury, methylation, *hgcAB*, microbial community, biofilm, organic matter, diffusion, dynamics

## Abstract

Understanding contaminant dynamics in ecosystems requires considering interactions between habitats—an aspect often overlooked in research. Mercury (Hg) studies typically focus on methylmercury (MeHg) production in sediments, often neglecting the role of biofilms such as periphyton. This study analyzes sediments and periphyton in a disturbed river using biogeochemical and metagenomic approaches. We found that microbial communities differed between habitats, but Hg-methylating microbes were taxonomically similar, with higher abundance in sediments. Organic matter (OM), a key Hg vector, likely affects Hg dynamics differently across habitats: MeHg concentrations increased with increasing terrigenous OM in sediments, whereas in periphyton, MeHg increased with greater contributions of aquatic-derived OM. Surprisingly, periphyton showed higher MeHg concentrations than sediments, despite lower *hgcA* abundance, the gene associated with MeHg production. Our multi-indicator analysis provides a conceptual model suggesting that MeHg is primarily produced in active sediments (indicated by elevated carbon dioxide and methane), diffuses into the water column (supported by carbon dioxide-MeHg correlations), and accumulates in protein-rich periphyton in shallow, low-flow waters where prolonged exposure can enhance MeHg retention. While some MeHg production occurs in periphyton, especially at a wetland site with thick growth, periphyton at a hydroelectric-impacted site showed the highest MeHg levels despite absent *hgcA* and methylation activity, pointing towards MeHg retention from the water. As a major food source for primary consumers, periphyton likely redistributes accumulated MeHg through the food web. This study highlights the importance of considering MeHg transfer between habitats and the need to examine entire aquatic ecosystems to fully understand MeHg dynamics.

## Graphical Abstract

**Graphical Abstract:**
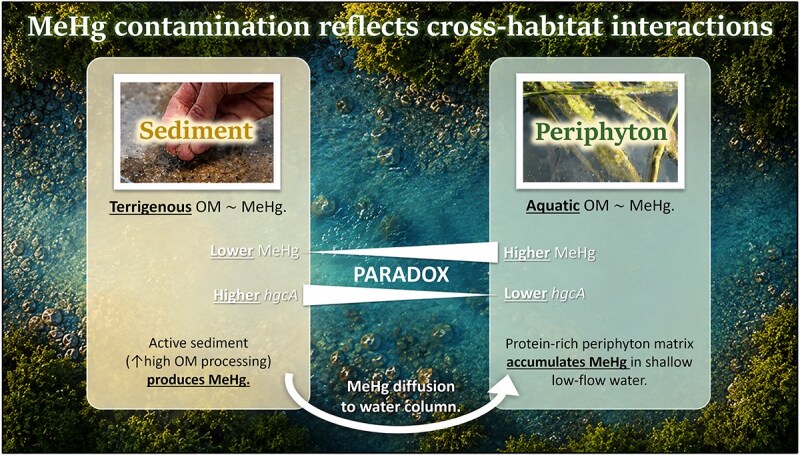

## Introduction

Mercury (Hg), found in minerals and coal, is released into the environment through mining, fossil fuel burning, and natural events like volcanic eruptions and forest fires [1]. Hg can travel long distances through air currents and water and deposit far from its source [2]. A major health concern is its transformation by microorganisms into toxic methylmercury (MeHg), which bioaccumulates in organisms and biomagnifies in food chains [3, 4]. Humans are exposed mainly through consumption of contaminated fish, leading to potential neurological and developmental consequences [5].

Hg methylation, the conversion of inorganic Hg to MeHg, is a crucial process that precedes contamination of trophic webs [6]. This transformation occurs in anoxic environments rich in organic matter (OM) that support anaerobic metabolism like freshwater sediments, wetlands, and flooded soil [7–11]. Aquatic biofilms like periphyton, a diverse community of algae, bacteria, and other microorganisms adhering to submerged surfaces [12], play an important role in Hg methylation [13–16]. While periphyton has historically received less attention than sediment as methylation hotspot [17, 18], recent studies emphasize their significance and the need to consider their connectivity to other environmental compartments when evaluating MeHg dynamics [17].

Hg methylation is primarily performed by anaerobic microbes, specifically bacteria and archaea with the *hgcAB* genes [10, 19]. However, the transformation of Hg to MeHg offers no detoxification benefit to the microbes, and *hgcAB* expression is not regulated by Hg levels [20, 21]. The original function of *hgcAB* is unknown, and Hg methylation likely occurs co-metabolically as an accidental side reaction during other processes advantageous to the *hgcAB* host [22]. Thus, while *hgcAB* is essential for Hg methylation, its presence or expression does not necessarily correlate with methylation rates or MeHg levels [11, 22–24].

Instead, Hg methylation is modulated by a range of environmental factors, including redox conditions, OM quantity and quality, and microbial community structure [7, 25–27]. Anoxic environments with ample and easily degradable OM and the presence of specific anaerobic microbial guilds, such as sulfate reducers, methanogens, fermenters, iron reducers, acetogens, and syntrophs, are recognized as favorable environments for Hg methylation [6, 28–30]. Understanding how environmental conditions influence microbial communities across habitats such as sediments and periphyton is essential, as these communities, in turn, regulate Hg methylation [27]. Notably, periphyton can exhibit methylation rates similar to or even higher than those of sediments in some environments, emphasizing the need to consider the entire ecosystem [13, 14].

Furthermore, disturbances in watersheds affect Hg methylation conditions differently in periphyton and sediments [31]. Periphyton microbial communities are more sensitive to changes in light and water flow [32], while sediment communities are influenced by OM sedimentation and redox conditions [31]. Land use changes like deforestation and wildfires can increase soil erosion and runoff, bringing more OM into aquatic systems, stimulating microbial activity, and enhancing Hg availability for methylation, as Hg is typically bound to organic-rich particles [31, 33–41]. Wildfires also release ash and char with elevated Hg levels, further boosting Hg inputs [42]. Additionally, hydrological changes, like impoundment from hydroelectric dams, can increase Hg input from soil and shift sediment redox conditions, promoting anaerobic microorganisms involved in Hg methylation [11, 31, 43].

Our study is part of a collaborative project between industry, academia, and Indigenous communities in Canada [44]. It was prompted by elevated MeHg levels in fish, reducing the amount of fish meals recommended five years after two hydroelectric dams were built on the St. Maurice River in Quebec, near the Atikamekw community of Wemotaci [45]. This river has faced multiple disturbances, including deforestation, a wildfire, and the creation of an artificial wetland for fish reproduction. Our research aims to address the root of MeHg contamination in this ecosystem by investigating MeHg production and fate. We conducted biogeochemical analyses to identify MeHg hotspots and used metagenomics to compare Hg-methylating microbial communities in sediments and periphyton, identified by the *hgcA* gene. We examined how environmental factors, particularly OM, drive MeHg input and production across the two habitats. Our study concludes with conceptual models on cross-habitat MeHg interactions, considering both sediments and periphyton as potential habitats for Hg methylators.

## Materials and methods

### Study site description

Our study examines a 40 km stretch of the St. Maurice River in Quebec, a boreal forest area impacted by watershed disturbances (Supplementary Fig. S1). Upstream, Wemotaci “WEM” sites are man-made wetlands built in 2008 to expand fish reproduction habitats, featuring shallow, stagnant water dominated by aquatic plants (macrophytes). Midstream, Chute-Allard “CA” sites are located within the impoundment zone of a hydroelectric dam from 2008, near a 200 km^2^ wildfire burn area from 2010. About 30 km downstream, Rapides-des-Coeurs “RDC” sites are also located in an impoundment zone of another 2008 hydroelectric dam, influenced by logging. Unlike WEM sites, CA and RDC sites are riverine environments with fewer aquatic plants. Despite these differences, all sites share the key feature of shallow, slow-flowing waters where periphyton and sediments coexist in close proximity—conditions that support our investigation on the interactions between these two habitats.

### Sampling

Sampling was conducted (as described in Supplementary Material S2) in August 2017, nine years after the construction of the two hydroelectric run-of-river dams and the artificial water channels (Supplementary Fig. S1). WEM samples came from constructed wetland channels, while CA and RDC samples were from sediments or soils flooded next to dam construction and impoundment. Sediment samples were collected as undisturbed cores to preserve the vertical structure, with the upper 10 cm analyzed to distinguish sediment depth for both chemical and metagenomic analyses. Periphyton was sampled from wood branches and macrophytes. Water was sampled using a peristaltic pump and a groundwater filtering system (0.45 μm pore size). All samples were stored for chemical analysis (−20°C) and metagenomic sequencing (−80°C).

### Chemical analyses

THg, MeHg, OM content, C/N ratio, major cations, anions and trace elements, DOC, dissolved CO_2_ and CH_4_, phosphorous, nitrogen species, dissolved oxygen, and pH were measured as described in Supplementary Material S3.

### Metagenomic analyses

Sediment and periphytic DNA were extracted and shotgun sequenced as described in Supplementary Material S4. Reads were trimmed (Trimmomatic) and taxonomically profiled using SingleM by clustering rplB (ribosomal protein L2) marker gene sequences into operational taxonomic unit (OTU)-like sequence clusters derived from shotgun metagenomes [46]. A complementary metagenome-assembled genome approach was undertaken in Lawruk-Desjardins et al. [47], where genome-resolved analyses provide independent support for, and further expand upon, the functional and taxonomic patterns reported here. Trimmed reads were assembled (Megahit) [48] and annotated (JGI IMG/M) [49]. Amino acid sequences were screened for HgcA with an hmm model [10]. HgcA sequences were taxonomically assigned (blastp in diamond) using GTDB [50]. *HgcA* gene abundance was normalized by dividing gene coverage by the total number of reads per metagenome, reported as *hgcA* copies per 1 million metagenomic reads. A detailed description is provided in Supplementary Material S4.

### Data treatment and visualization

Mean values of MeHg, MeHg/THg, THg, and *hgcA* were compared using the nonparametric Mann–Whitney rank sum test between the two habitats per site. Non-metric multidimensional scaling (NMDS) and redundancy analysis (RDA) were performed on a Bray–Curtis dissimilarity matrix of Hellinger-transformed OTUs. Significant differences of the diversity indices were assessed by Mann–Whitney U test. The proportion of *hgcA*+ taxa in each habitat was expressed as a percentage of total *hgcA* abundance per habitat. Significant differences in environmental parameters were assessed using ANOVA followed by a *post-hoc* Tukey’s Honestly Significant Difference (HSD) test. Significant linear regression models of MeHg, THg, or MeHg/THg ratio against C/N ratio or OM content were checked for normality and homoscedasticity before plotting. More details are given in Supplementary Material S5.

## Results and discussion

### MeHg concentrations and *hgcA* abundance

**Figure 1:**
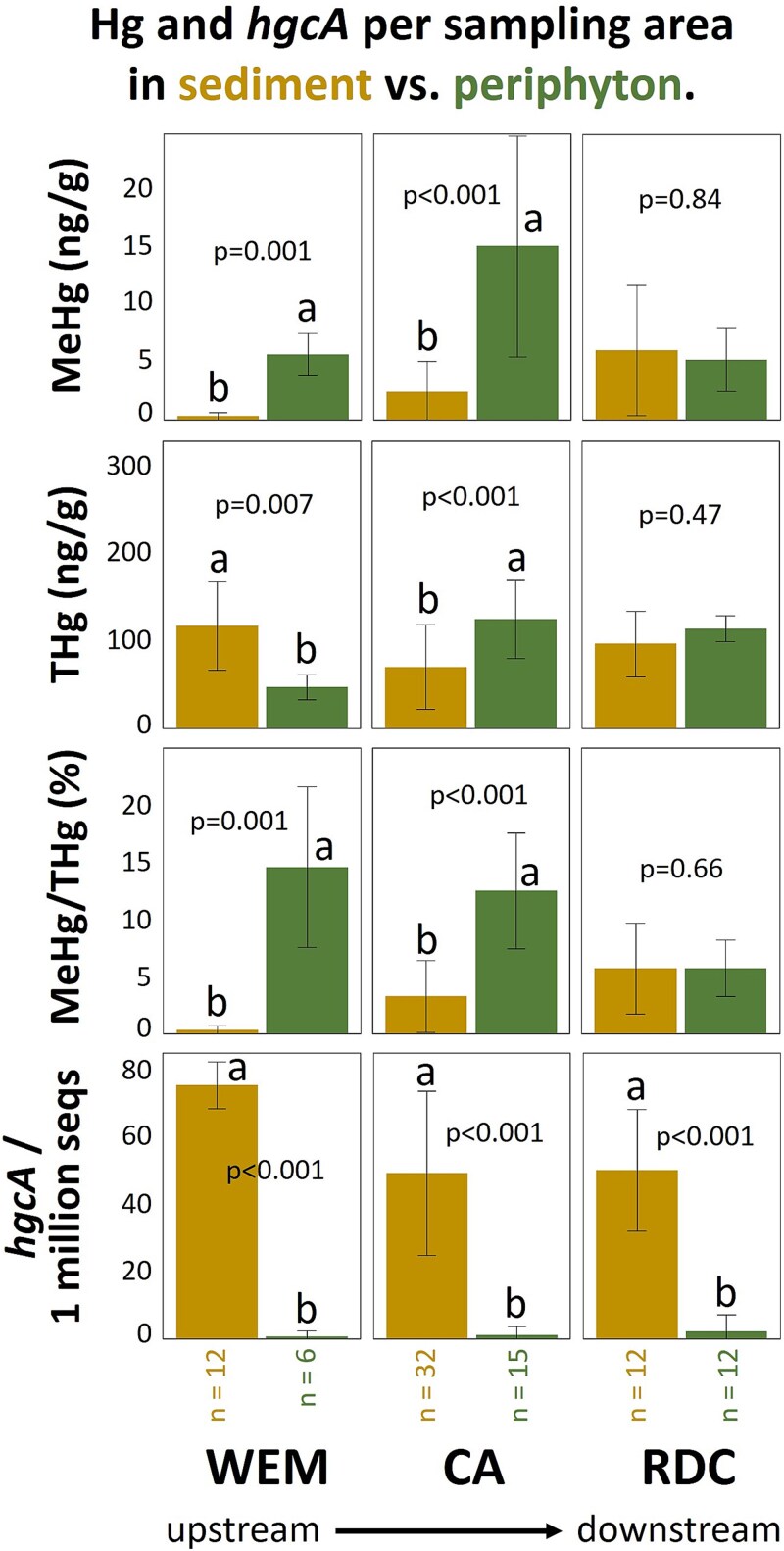
Mean ± standard deviation of MeHg and THg concentrations, along with the MeHg/THg ratio, and hgcA abundance in sediments (brown) and periphyton (green) across the three sampling areas: WEM, CA, and RDC. The number of samples (n) is provided below each bar. Significant differences between sediment and periphyton were assessed using the Mann–Whitney rank sum test performed separately for each site and are denoted by different letters.

We investigated periphyton and sediments as potential Hg methylation hotspots within the disturbed river, measuring MeHg, total Hg (THg), and the MeHg/THg ratio across our three sampling areas (WEM: constructed wetlands, CA: hydroelectric impoundment zone impacted by wildfire, RDC: hydroelectric impoundment zone impacted by deforestation). Results showed that each habitat contributes to MeHg contamination in the river, with variations in sediment and periphyton among sites (Fig. 1).

MeHg is higher in periphyton than sediments at WEM and CA, while RDC shows similar concentrations across habitats. Average MeHg concentrations are 3 ± 4 ng/g in sediments and 9 ± 8 ng/g in periphyton, falling within typical reported ranges [11, 14, 15, 24]. Periphyton at CA sites have the highest MeHg concentrations (15 ± 10 ng/g), possibly due to the combined effects of increased Hg input and methylation from wildfire and dam impacts [11, 31, 42].

THg is higher in sediments at WEM, while CA has higher THg in periphyton. RDC shows similar THg concentrations in both habitats. No significant difference in THg levels between sediments and periphyton was found overall.

The MeHg/THg ratio, a proxy for Hg methylation [11, 51], is four times higher in periphyton than in sediments. This difference is especially pronounced at the WEM and CA sites, while the ratio is similar at RDC. However, the reliability of this ratio as an indicator for methylation in our study is uncertain, as it does not consistently correlate with methylation activity in the literature and can be influenced by various environmental, seasonal, and spatial factors, such as the inputs of Hg-rich OM from the watershed [52, 53].

We also examined *hgcA* gene abundance, essential for MeHg production. *HgcA* was consistently higher in sediments at all sites, with 55 ± 23 *hgcA*/million seqs in sediment compared to 3.3 ± 4 in periphyton. This indicates that sediment environments may play a more significant role in MeHg production. This difference is particularly pronounced in the WEM area, where *hgcA* abundance in sediments surpasses that in periphyton by a factor of 90. Although the presence or expression of *hgcAB* does not always correlate with methylation rates or MeHg levels in various studies, *hgcAB* remains crucial for Hg methylation [11, 22–24]. In our study, the observation of higher MeHg concentrations in periphyton despite higher *hgcA* abundance in sediments represents a central paradox that motivated further investigation into the underlying processes.

### Hg methylators within the overall microbial community composition

**Figure 2:**
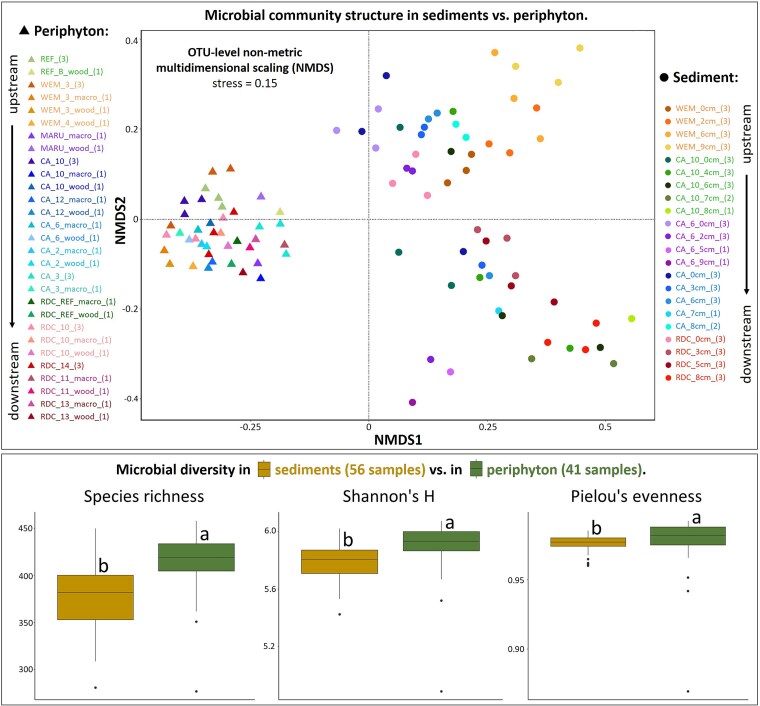
Microbial community structure in sediments vs. periphyton. The upper panel presents the β-diversity (between-sample diversity) as OTU-level NMDS of all periphyton (triangles) and sediment (dots) samples. The legends for periphyton and sediment samples are on the left and right margins, respectively. In the legends, samples are ordered from upstream to downstream of the river. The lower panel presents the α-diversity (within-sample diversity) as three different diversity indices for all sediment (brown) and periphyton (green) samples, respectively pooled. Significant differences were assessed by the Mann–Whitney U test and are denoted by letters.

To explore Hg methylation, we examined microbial communities, finding distinct clustering between sediment and periphyton samples in an NMDS, indicating important compositional differences (Fig. 2, upper  panel). These clusters likely reflect varying environmental influences: periphyton is shaped by plant exudates, light and nutrient availability [32, 54], while sediments are influenced by particle composition, OM content, and redox conditions [31]. These factors can influence microbial activities and Hg dynamics differently in each habitat [55–57].

Sediments show higher between-sample diversity than periphyton (Fig. 2, upper panel; Supplementary Figs S7 and S9), likely reflecting dispersal limitations in deeper sediment layers and greater environmental heterogeneity [58]. Periphyton communities are more cohesive, probably due to extensive water and nutrient connectivity, as well as higher metabolic autonomy as primary producers [59, 60]. The WEM sediment community differs from others, likely because it was once subsoil, stripped of topsoil during excavation, and turned into sediment in artificial water channels, whereas other sites are river sediments or flooded soils. Additionally, the WEM site’s OM is mainly produced by dense macrophyte growth, while OM at other sites is transported from the watershed. These qualitative patterns are further examined quantitatively in the RDA analyses (Supplementary Fig. S15), which explore the influence of geochemical variables on microbial community composition. Additional details are provided in Supplementary Material S6 and Supplementary Fig. S8).

Periphyton shows greater within-sample diversity (Fig. 2, lower panel), as its structural heterogeneity supports a wider range of microorganisms [60].

**Figure 3:**
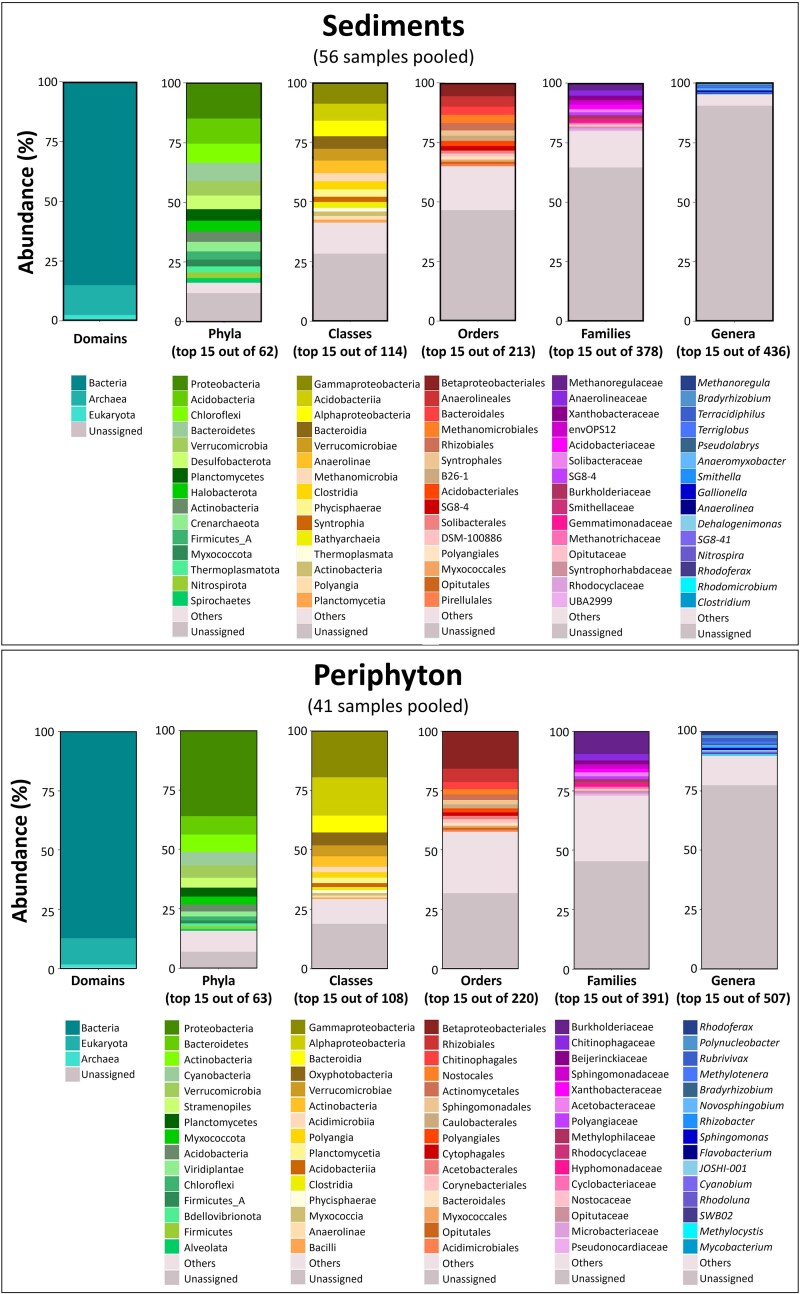
Most abundant taxa in sediments and in periphyton for all samples pooled together, respectively. The bar plots are the relative abundance of taxonomic groups at various taxonomic levels, color-coded according to the taxa legend in the lower part of the figure. The arrows indicate taxa affiliation at varying taxonomic levels.

Analysis of dominant taxa shows that periphyton contains more eukaryotes but fewer archaea than sediment (Fig. 3), consistent with its lower *hgcA* abundance (Fig. 1), as only bacteria and archaea encode this gene [10]. Several dominant phyla include known Hg methylators (Supplementary Material S10). Cyanobacteria, abundant in periphyton but absent among top sediment taxa, do not methylate Hg but typically thrive in periphyton [61]. Their high prevalence, along with eukaryotes, suggests greater light and oxygen availability in periphyton compared to sediment, potentially supporting photic and biotic MeHg demethylation via mostly aerobic *merAB*-harboring microbes [62]. However, a prior analysis of periphyton from the same sampling area found no *merB* and low *merA* levels, suggesting limited, likely abiotic demethylation, consistent with higher MeHg levels in periphyton [15]. Sediments, with limited light and more anaerobic microbial communities typically lacking the *mer* operon [63], likely exhibit even lower demethylation rates than periphyton.

Consistent with NMDS results (Fig. 2), sediment taxa show site-specific patterns at the phylum level, unlike periphyton (Supplementary Figs S11 and S12). Sediment samples also reveal depth-related trends, such as archaea increasing with sediment depth at the WEM sites, likely because archaea can thrive in anoxic environments with less nutrients and lower pH, which are often found deeper in sediments or soils. In contrast, bacteria typically dominate OM-rich upper soil and sediment layers [64]. Topsoil removal during WEM water channel construction may have contributed to higher archaeal abundance there. *HgcA* levels are highest at WEM (Fig. 1), suggesting a higher proportion of *hgcA* hosts within the archaeal community than within the bacterial community. However, lower MeHg concentrations at WEM imply that archaea may be less effective Hg methylators than bacteria. This aligns with recent studies suggesting differential *hgcAB* regulation—by an *arsR*-like regulator in bacteria [22], and a toxin-antitoxin system in archaea [65]. An alternative explanation for the low MeHg levels despite high *hgcA* abundance at WEM is increased MeHg efflux from sediment into the overlying water. A recent study reported higher MeHg fluxes from sediments to water in macrophyte-rich, OM-rich, anoxic sediments [66], aligning with the greater macrophyte abundance at WEM compared to other sites [15].

The relatively large fraction of unassigned reads reflects the high confidence thresholds used for marker-gene-based taxonomy (≥96.7% identity) [46]. Reads that do not meet this criterion remain unassigned. These thresholds provide robust and consistent taxonomic profiling without relying on assembly or binning. The sediment taxonomic patterns reported here are further supported by genome-resolved analyses in a related metagenome-assembled genome-based study [47].

**Figure 4:**
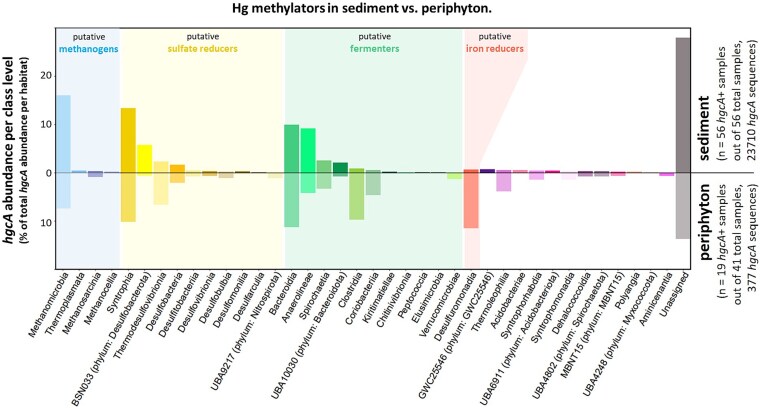
Relative abundance of hgcA-containing taxa per habitat at class level in sediment vs. periphyton. Taxonomic classes are arranged and color-coded based on their presumed metabolism.

Following the analysis of the overall microbial community, we examined contigs containing the *hgcA* genes (hereafter referred to as *hgcA*+ taxa; Fig. 4) and taxonomically identified putative methanogens, sulfate reducers, fermenters, and iron reducers—all groups known to include Hg methylators (Supplementary Material S13) [10, 29, 30]. Genome-resolved analysis of *hgcA*+ metagenome-assembled genomes from Lawruk-Desjardins et al. [47] support these assignments and confirm that these guilds indeed harbor *hgcA* genes. In both habitats, dominant *hgcA*+ groups are consistent: Methanomicrobia (methanogens), Syntrophia (sulfate reducers), Bacteroidia (fermenters), and Desulfuromonadia (iron reducers), which aligns with findings from the parallel study focusing on *hgcA*+ metagenome-assembled genomes from these sites [47]. Although *hgcA* hosts are less abundant in periphyton (Fig. 1), their community composition closely mirrors that in sediments (Fig. 4), suggesting possible microbial exchange. This may be driven by the close proximity of sediments (<2 m below periphyton) and low water flow, particularly at WEM.

### Impact of environmental factors on microbial communities

RDA relating microbial community structure to environmental parameters (Supplementary Material S5) was performed using MeHg, THg, MeHg/THg, OM%, C/N ratio, various metals and elements (Na, Mg, Al, K, Ca, Sc, Cr, Mn, Fe, Co, Ni, Cu, Zn, V, Se, Sr, As, Mo, Y, Ag, Cd, Sb, Ba, Eu, La, Ce, Pr, Nd) in sediments, and additional aqueous variables like temperature, pH, oxygen, DOC, CO_2_, CH_4_, Cl^−^, F^−^, NO_3_^−^, SO_4_^−^, and nutrients. The RDA revealed that only 4% of sediment microbial community variation is explained by the measured variables, with MeHg, As, and C/N showing significant correlations (Supplementary Material S14; Supplementary Fig. S15).

MeHg concentrations in sediments are lower at WEM and higher at RDC. Despite low MeHg levels, WEM sediments exhibit the highest *hgcA* abundances and dense macrophyte growth, which may facilitate MeHg release from sediments into the water column [66]. In contrast, RDC sediments have minimal macrophyte cover and show increasing MeHg with sediment depth, indicating stronger MeHg retention in the RDC sediments.

The taxonomic composition of microbial communities at WEM correlate with elevated As levels, which increase with sediment depth (Supplementary Fig. S15). As was also a significant predictor of the metagenome-assembled genome-based community structure in a related genome-catalogue study conducted at the same sites [47]. Recently, As was found to regulate *hgcAB* expression via an upstream co-transcribed *arsR* gene [22]. This supports the co-occurrence of high As levels and highest *hgcA* abundances observed at WEM (Fig. 1).

The C/N ratio indicates OM origin, with higher values reflecting terrigenous inputs and lower values indicating aquatic sources, and is known to influence microbial communities [67]. CA_6 sediments show the highest C/N ratios, consistent with terrigenous OM accumulation from hydroelectric dam disturbances [11, 45]. In contrast, WEM sediments have the lowest C/N ratios, likely due to carbon-rich topsoil removal and dominance of nitrogen-rich aquatic OM from macrophytes and periphyton. This suggests WEM sediments are probably largely composed of periphyton and macrophyte debris, which should result in high MeHg concentrations, given the elevated MeHg levels observed in WEM periphyton. However, WEM sediments have the lowest MeHg concentrations, once again pointing to increased MeHg diffusion from sediment to the water, likely facilitated by the high abundance of macrophyte roots that alter sediment properties, making them favorable for element solubility and diffusion [66]. The observed pattern—low C/N ratios and low MeHg at WEM, and high C/N ratios and high MeHg at CA—is consistent with findings from Bravo et al. [7]. The flooded topsoil at CA from hydroelectric dam impoundment creates OM- and Hg-rich anoxic conditions highly favorable for Hg methylation [43].

Periphytic microbial communities show weak correlations with environmental parameters, with only 1% of variation explained by aqueous and periphytic chemistry (Supplementary Fig. S16). This likely reflects lower between-sample variability and greater metabolic self-sufficiency—the ability of these communities to internally cycle nutrients and sustain essential functions independently of external inputs [68, 69]. While no clear spatial patterns emerged, MeHg in periphyton and DOC in water were significantly correlated. Both are low at WEM, suggesting reduced terrigenous OM and lower Hg and MeHg exposure. High periphyton biomass at WEM may also biodilute MeHg, explaining lower concentrations than at CA [70].

### OM as a driver for MeHg

**Figure 5:**
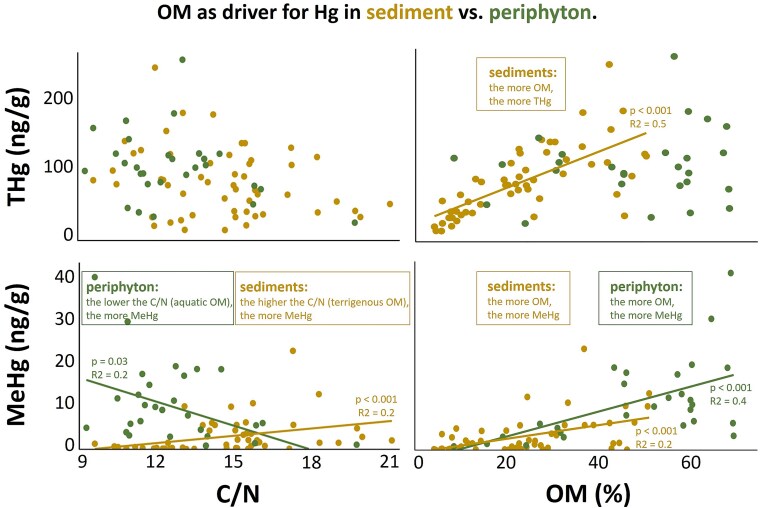
OM quality (C/N) and quantity (OM %) as drivers for Hg in sediment (brown) and periphyton (green). Significant correlations among THg, MeHg, C/N, and OM are indicated by a regression line, accompanied by p and R^2^ values.

Given the minor but significant influence of C/N and DOC on microbial communities (Supplementary Figs S15 and S16), we examined how OM content (OM%) and quality (C/N) affect MeHg dynamics. Sediments have significantly higher C/N ratios (15 ± 3) than periphyton (12 ± 3), indicating more terrigenous OM, consistent with watershed disturbances. Periphyton’s lower C/N suggests more aquatic, self-produced OM. MeHg and MeHg/THg ratios in both habitats positively correlate with OM% (Fig. 5; Supplementary Fig. S18), suggesting OM enhances microbial activity and co-metabolic Hg methylation, regardless of OM origin. Further details are provided in Supplementary Material S17.

The source of OM (C/N ratio) affects MeHg concentrations differently across habitats: in sediments, higher C/N ratios (more terrigenous OM) correlate with elevated MeHg levels (Fig. 5), consistent with Bravo et al. [7] and Millera Ferriz et al. [11]. Bravo et al. [7] reported high MeHg in terrigenous OM-rich sediments with low methylation rates and low MeHg in aquatic OM-rich sediments with high methylation, suggesting watershed-derived MeHg as the main source in terrigenous sediments. However, this pattern may also reflect enhanced MeHg fluxes from aquatic OM-rich sediments to the water, often associated with dense macrophytes that can enhance MeHg diffusion [66]. Thus, OM source may shape sediment MeHg patterns by influencing not only MeHg input and production as suggested in Bravo et al. [7], but also its export.

While sediments show a positive correlation between C/N ratios and MeHg, periphyton exhibits a negative correlation, with lower C/N ratios linked to higher MeHg concentrations (Fig. 5). In periphyton, low C/N may signal (i) high biomass productivity potentially leading to increased methylation activity—especially plausible in the high aquatic-biomass-producing WEM water channels but less likely at the CA and RDC sites where macrophyte growth is sparse—and (ii) high protein content that can enhance MeHg accumulation, as MeHg preferentially associates with protein-rich matrices [71–73]. Although *hgcA* abundance in periphyton is low, methylation activity may still be mediated by low-abundance but transcriptionally active populations, a possibility that cannot be evaluated here in the absence of metatranscriptomic data, and together, these mechanisms help explain why periphyton can accumulate more MeHg than sediments [74]. Elevated MeHg concentrations in periphyton are unlikely to result from reduced demethylation. Instead, periphyton is generally expected to experience greater overall demethylation potential than sediments due to increased light exposure favoring abiotic demethylation and the presence of aerobic microbial communities carrying the *mer* operon [62]. However, a previous analysis of periphyton from the same system found no *merB* and low *merA* abundance, suggesting that biotic demethylation via the *mer* operon is limited and that abiotic processes likely dominate [15]. Together, these observations indicate that high MeHg in periphyton is more consistent with enhanced accumulation and/or methylation rather than reduced demethylation. High periphytic MeHg accumulation has important food web implications, since periphyton is a key MeHg entry point as an important food source for primary consumers [69, 73, 75].

### Sediments and periphyton as key players in MeHg production and accumulation

**Figure 6:**
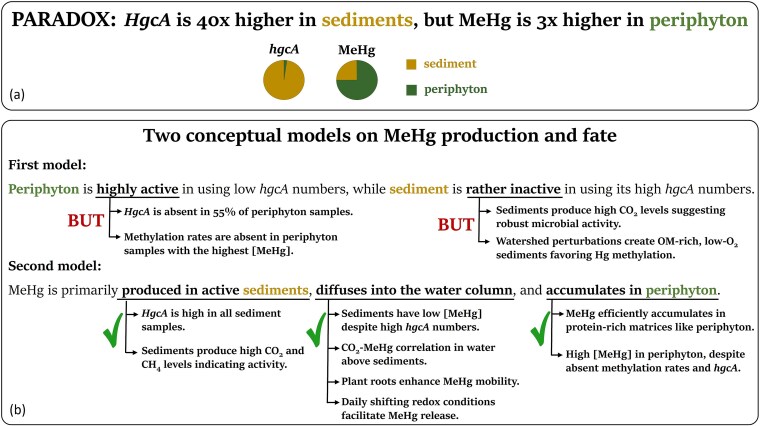
(a) Paradox: the abundance of the *hgcA* gene (per 1 million sequences), essential for MeHg production, is 40× higher in sediments (brown) than in periphyton (green), but periphyton has 3× more MeHg (ng per g of matrix). (b) Summary of the two conceptual models for MeHg production and fate, with the main data and arguments that support or challenge each model.

Our study lays the groundwork for understanding MeHg production and fate in the river ecosystem. Microbial communities differed between habitats, whereas Hg-methylating taxa were taxonomically similar but more abundant in sediments. OM showed habitat-specific associations with MeHg, with terrigenous OM linked to higher sediment MeHg and aquatic OM linked to higher periphytic MeHg. Based on 56 sediment and 41 periphyton samples, MeHg levels are about 3 times higher in periphyton, even after normalizing to carbon. Paradoxically, the *hgcA* gene—essential for MeHg production—is 40 times more abundant in sediments, despite their lower MeHg concentrations (Fig. 6a).

This discrepancy between MeHg concentrations and *hgcA* abundance led to consider two conceptual models describing MeHg dynamics in our river ecosystem (Fig. 6b). The first model suggests that periphyton may be more active in Hg methylation than its low *hgcA* levels imply, while sediments may be less active despite high *hgcA* abundance. Under this scenario, a substantial fraction of *hgcA* in sediment may not be expressed or may serve functions unrelated to MeHg production. Since *hgcAB* expression is not Hg-regulated [20, 21] and its function remains unclear [22], *hgcAB* abundance does not consistently correlate with MeHg concentrations [11, 22–24]. Notably, this mismatch can occur across diverse environments and is not limited to sediments alone.

The literature generally highlights high microbial activity in periphyton—driven by factors such as oxygen, light, and nutrient availability—and emphasizes the role of hypoxic niches in Hg methylation [68, 76]. However, sediments are also dynamic and metabolically active environments. In our study, elevated CO_2_ production in sediments from our river section indicates robust microbial activity (Supplementary Fig. S19) [45]. This activity likely results from constant OM input: aquatic OM from macrophyte and periphyton debris at WEM, and terrigenous OM from watershed perturbations at CA and RDC. Such OM-rich, low-oxygen sediments are generally favorable for anaerobic Hg-methylating microorganisms.

At WEM, high *hgcA* abundance coincides with high As concentrations in sediment (Supplementary Fig. S15), which may support active *hgcA*+ populations, as *hgcAB* and *arsR* are co-regulated by As [22]. Periphyton can also develop localized anoxic microzones that may be favorable to methylators, particularly at WEM with thicker periphyton growth [15, 77]. However, WEM periphyton does not exhibit the highest MeHg concentrations compared to thinner biofilms at CA and RDC (Fig. 1). This may reflect potential MeHg biodilution within the dense biomass at WEM [70]. Collectively, these observations provide limited support for the first conceptual model, which is further undermined by the extend of the 40-fold higher *hgcA* levels in sediments (Fig. 6a) and by the complete absence of *hgcA* in 55% of periphyton samples (Fig. 4). Notably, *hgcA* was absent in periphyton samples with the highest MeHg concentrations (Supplementary Fig. S21; Supplementary Material S20), raising the possibility of unknown methylation pathways independent of *hgcAB*. However, this seems unlikely given (i) the well-established necessity of *hgcAB* presence for MeHg production and (ii) the implausibility of such pathways being exclusive to periphyton. A more likely explanation is that MeHg in periphyton originates in large parts from external sources, prompting a second model on MeHg dynamics.

The second conceptual model, therefore, proposes that MeHg is primarily produced in active sediments, diffuses into the water column, and accumulates in the protein-rich periphyton matrix, which efficiently retains contaminants (Fig. 6b) [74, 78]. Submerged in shallow, low-flow waters above the sediments, periphyton is well-positioned to absorb MeHg. This model is consistent with higher periphytic MeHg at CA than WEM (Fig. 1), despite higher periphytic methylation rates at WEM and largely undetectable methylation rates at CA reported previously [15].

At WEM, sediment-to-water MeHg diffusion appears plausible, because (i) sediment MeHg is lowest despite highest *hgcA* abundance (90-fold higher than in periphyton) (Fig. 1), (ii) MeHg-rich periphyton debris (indicated by low C/N in sediment, Supplementary Fig. S15) would be expected to elevate sediment MeHg but does not (Fig. 1), and (iii) abundant macrophyte roots may enhance solute mobility by altering sediment properties [66]. Low MeHg in these active, *hgcA*-rich sediments is unlikely to result from greater MeHg demethylation in sediments compared to periphyton, as sediment conditions are generally less favorable for demethylation [62], and measured MeHg likely reflects methylation-demethylation equilibrium.

The conceptual model of MeHg flux from sediments is further supported by (i) low sediment MeHg at CA and WEM despite high microbial activity in OM-rich sediments, inferred from elevated CO_2_ and CH_4_ in overlying waters (Supplementary Fig. S19), (ii) significant CO_2_-MeHg correlations in water [44, 45], and (iii) redox oscillations known to promote MeHg mobilization from sediments [79–82] (Supplementary Material S22, Supplementary Table S23).

This MeHg flux from sediments, combined with shallow, low-flow water at our sites, likely enhances MeHg absorption by periphyton’s protein-rich matrix conductive to MeHg accumulation [71–73], explaining higher MeHg in periphyton than in sediments at WEM and CA (Fig. 1). At WEM, elevated MeHg in periphyton likely originates from both *in situ* production in thick biofilms and uptake of sediment-derived MeHg, whereas at CA, it appears mostly sediment-derived, given low or absent methylation rates and *hgcA* in CA periphyton [15], and active *hgcA*-rich sediments acting as traps for watershed-derived OM and THg [11].

At RDC, periphytic MeHg concentrations are similar to those in sediments—the only site where this occurs (Fig. 1). Unlike other sites, MeHg increases with sediment depth at RDC (Supplementary Fig. S15), and diffusion may be limited by sparse macrophytes [66] and possibly oxygenated tributary water [81]. These conditions likely promote MeHg retention in sediments, reducing its availability to periphyton. Thus, periphytic MeHg at RDC may reflect a greater contribution from watershed inputs and limited *in situ* production rather than sediment diffusion.

These spatial variations in MeHg flux illustrate the limitations of using MeHg/THg ratios as methylation proxy at small scales. Past watershed disturbances may have redistributed THg across habitats, causing local concentration shifts that confound such proxies [78]. MeHg/THg ratios may be more reliable in large-scale, stable, or controlled environments. Other methods to assess Hg methylation, such as *hgcAB* abundance or *in situ* methylation rates, are limited by spatial and temporal variability [24, 83]. A more comprehensive understanding requires multiple indicators. Promising tools include carbon (MeHg) and Hg isotope ratio analysis to trace MeHg cycling across environmental matrices [84], and MeHg diffusion measurements using sediment-inserted dialysis chambers [81].

Our data interpretation of MeHg fluxes from sediment to periphyton is consistent with studies reporting MeHg diffusion from sediments into the overlying water and periphyton layers [66, 85], although flux direction can vary among ecosystems [16, 81]. Notably, we observed elevated MeHg in periphyton even in the absence of detectable *hgcA* genes or evidence of *in situ* methylation [15], reinforcing the view that periphyton in this system primarily functions as an MeHg accumulator rather than a site of production. In our impacted river system, MeHg contamination appears to emerge from complex interactions across habitats: soils likely serve as the main source of Hg and MeHg inputs, sediments act as the dominant methylation zones, and periphyton facilitates the entry of MeHg into aquatic food webs. By directly comparing sediments and periphyton as potential methylation hotspots, our study underscores the value of integrating multiple indicators and considering cross-habitat fluxes to better understand ecosystem-scale MeHg dynamics.

Given periphyton’s pivotal role as a MeHg reservoir and vector to primary consumers, bioremediation strategies involving MeHg-demethylating algae-periphyton consortia may hold promise for mitigation [85]. In parallel, watershed management approaches aimed at reducing soil erosion and OM runoff could help curb THg influx and, consequently, MeHg production.

## Supplementary Material

Supp_Material_Storck_et_al_2026_ycag176

## Data Availability

Metagenomic data is available through the European Nucleotide Archive under accession number PRJEB67532 for sediments and at IMG/M under GOLD study ID Gs0140997 for periphyton.
