# Supplementary material for: Cross-habitat interactions drive methylmercury contamination in a disturbed river ecosystem: novel metagenomic and biogeochemical insights
Source: ISME Commun. 2026 Jun 23;6(1):ycag176. doi: 10.1093/ismeco/ycag176 (PMC13362967; doi:10.1093/ismeco/ycag176)
Supplement: Supp_Material_Storck_et_al_2026_ycag176 [file supp_material_storck_et_al_2026_ycag176.docx]

Supplementary Information

**Cross-habitat interactions drive methylmercury contamination in a disturbed river ecosystem: Novel metagenomic and biogeochemical insights**

Veronika Storck^1,2^, Dominic E. Ponton^2^, Charlène Lawruk-Desjardins^1^, Lise Millera Ferriz^2^, Maxime Leclerc^2^, Susanne Kraemer^1,3^, Dolorès Planas^4^, Marc Amyot^2^, David Walsh^1^*

^1^ Biology Department, Concordia University, Montreal, Canada

^2^ Département de Sciences Biologiques, Université de Montréal, Canada

^3^ Aquatic Contaminants Research Division, Environment and Climate Change Canada, Montreal, Canada

^4^ Département de Sciences Biologiques, Université de Québec à Montréal, Canada

*Corresponding author: walsh.david@concordia.ca


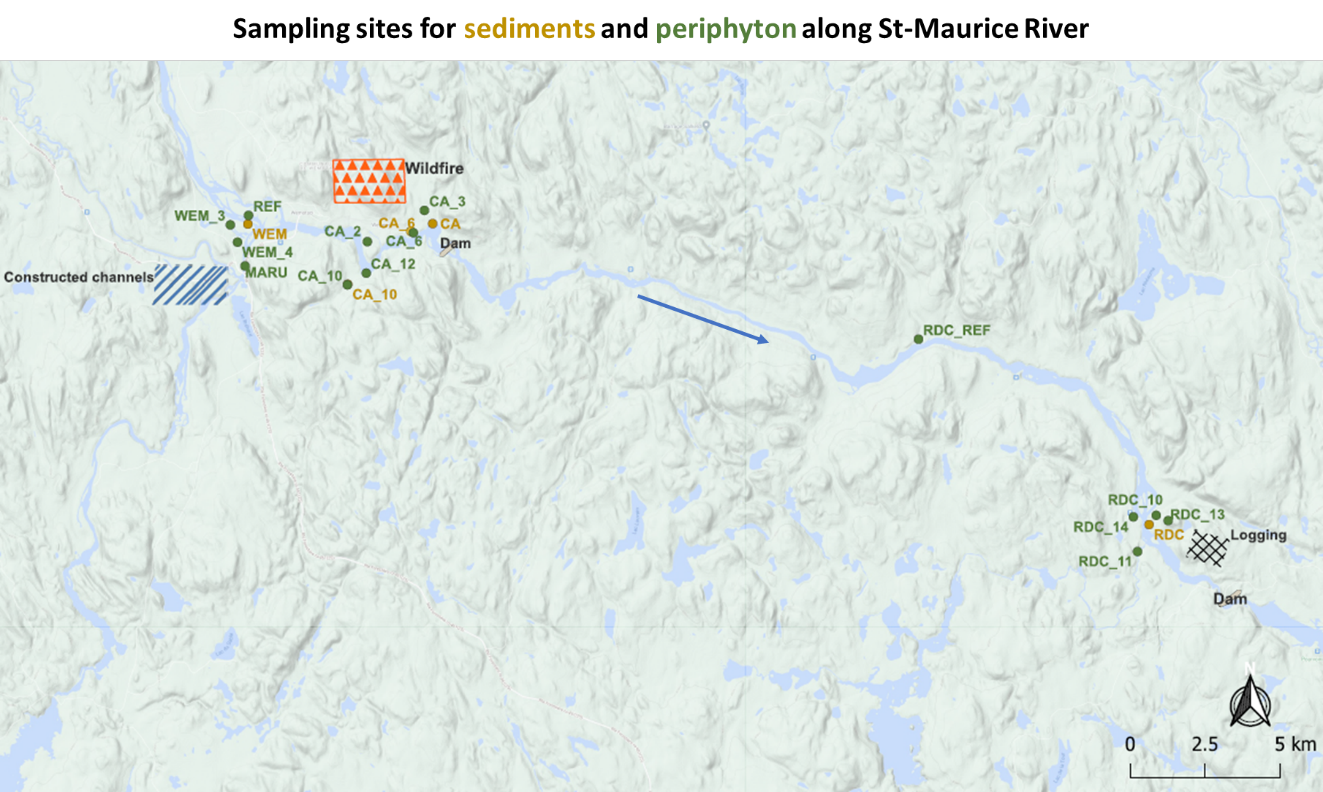


**Supplementary Figure S1.** Map of the sediment (brown) and periphyton (green) sampling sites. The St. Maurice River, stretching 560 km from Reservoir Gouin to the St. Lawrence River, flows through the Precambrian Shield in Quebec, Canada. Our study focuses on a 40 km section in Haute-Mauricie, near the Atikamekw reserve of Wemotaci. Two run-of-river hydroelectric plants, Chute Allard (CA) and Rapides-des-Coeurs (RDC), were established there in 2008. Designed to minimize flooding, CA and RDC pondages cover 2 km² and 3.7 km², respectively, leading to the loss of 2.4 km² of wetlands. To compensate, Hydro-Quebec created the WEM artificial wetlands upstream of CA, designed for wildlife conservation and yellow perch spawning. The river section experienced two more significant landscape perturbations: a 2010 wildfire affecting over 180 km² near CA and logging near RDC. Five years after dam construction, elevated MeHg levels in fish led to revised consumption guidelines (Ponton et al., 2021).

**Supplementary Material S2.** Water, sediment and periphyton sampling

Water temperatures ranged between 16 and 21°C.

Glassware for water sampling was acid-washed overnight with 5% HCl and 45% HNO_3_, and plasticware with 10% HCl. Both were rinsed with Milli-Q water and 0.45 µm filtered river water. Water samples were collected using a peristaltic pump and a groundwater filtering system with a 0.45 µm pore size. Sampling followed the ‘clean hands, dirty hands’ protocol for trace metal sampling (St. Louis et al., 1994).

Three undisturbed sediment cores per site were collected using a gravimetric hand corer. The cores were sealed, with overlying water jellified to preserve the water-sediment interface. In the lab, cores were cut and split, with one half sliced every cm for depth profiling. Subsamples were taken from four to five depths within the upper 0-10 cm sediment layer, with one to three replicates per depth. Samples were divided for chemical analysis (stored at -20°C) and metagenomic sequencing (stored at -80°C).

Natural substrates for periphytic biofilms included submerged wood branches and macrophytes. Pac-man boxes (0.68-liter) were used for sampling, modified from the 6-liter Downing box design (Downing, 1986). Care was taken to prevent contamination and material loss. Periphyton samples were collected with two to six replicates per sampling area, from both macrophytes and wood branches. Samples were stored at -20°C for chemical analysis, and at -80°C for metagenomic analyses.

**Supplementary Material S3.** Chemical analyses of sediment, periphyton and water

Sediment and periphyton samples were digested and analyzed for THg and MeHg as explained in Millera Ferriz et al. (2021). The digestate was analyzed for major cations and trace elements using ICP-MS/MS (Agilent 8900). OM content was determined by loss on ignition at 550°C, and C/N ratios were calculated using a CHNS-O Element Analyzer (Fisons EA-1108). Quality control included internal standards with recovery between 95-105%. For periphyton, precombusted filters were dried and combusted to determine dry weight and ash-free dry weight.

Water samples preserved with 0.04% HCl at 4°C were analyzed for THg using EPA 1631 with a Tekran 2600 cold vapor atomic fluorescence spectrometer (CVAFS). Samples were oxidized with bromine monochloride, reduced with tin chloride, and volatile Hg(0) was collected on a gold trap for detection. The detection limit was 0.04 ng/L. For MeHg, EPA 1630 was followed with a Tekran 2700 CVAFS after distillation. Sodium tetraethylborate formed volatile Hg species, separated by gas chromatography, and detected by CVAFS. Water DOC was measured using an Aurora 1030 TOC analyzer after digestion with phosphoric acid and persulfate. Dissolved gases (CO_2_ and CH_4_) were extracted from 100 mL water by shaking the samples using hydrocarbon-free air, and transferred to 12 mL vials for analysis. Phosphorus was measured using EPA 365.3 by forming a phosphoantimonylmolybdenum complex, reduced by ascorbic acid to heteroblue, with absorbance at 660 or 880 nm. Nitrogen was measured by converting all species to nitrate using persulfate at 120°C for 45 min, and then quantitatively reducing to nitrite by diazotizing with sulfanilamide and coupling with N-(1-naphthyl)ethylenediamine dihydrochloride, analyzed at 520 nm (Grasshoff et al., 2007). Cations and trace elements were analyzed with ICP-MS/MS (Agilent 8900) after preservation with 2% nitric acid and digestion (95°C with 3% HNO_3_ and 0.5% HCl for two hours). In-situ measurements of dissolved oxygen and pH were taken with a YSI Pro Plus multiparameter probe. Anions (Cl, F, NO_3_^-^, SO_4_^2-^) were measured by ion chromatography (Metrosep A Supp 5 – 150/4.0, Metrohm).

**Supplementary Material S4.** Metagenomic analyses

Sediment DNA was extracted from 250 mg of wet sediment using the PowerSoil DNA Isolation Kit (Mo Bio Laboratories Inc.). Periphytic DNA was extracted with the DNeasy PowerWater kit (Qiagen), including a 10-minute incubation at 65°C for cell lysis and 1 μL RNase treatment for DNA extraction. Shotgun DNA sequencing was performed using Illumina NovaSeq 6000 S4 PE150 at Genome Quebec. Raw metagenomic reads were quality-trimmed with Trimmomatic 0.32 and classified into OTUs and taxonomically assigned by profiling rplB for ribosomal protein L2 with SingleM (Woodcroft et al., 2024). OTUs were clustered at 97% identity. Reads were assembled using Megahit 1.0.6 (Li et al., 2016) and mapped to the assembly with BWA-MEM (Li and Durbin, 2010). Scaffolds were submitted to the JGI IMG/M for annotation (Markowitz et al., 2008). Protein sequences were screened for HgcA sequences with an hmm model (McDaniel et al., 2020), using a score threshold of ≥300. The score was set based on alignments against HgcA sequences from the reference database and identification of the more conserved motifs (NVWCAAGK, NVWCASGK, NVWCAGGK, NIWCAAGK, NIWCAGGK, or NVWCSAGK). HgcA sequences were taxonomically assigned with blastp in diamond using the GTDB (Parks et al., 2018). *HgcA* gene abundance was normalized by dividing gene coverage by the total number of reads per metagenome, reported as *hgcA* copies per one million metagenomic reads.

**Supplementary Material S5.** Data treatment and visualization

For comparing MeHg and *hgcA* between periphyton and sediment, mean values were calculated and compared using the Mann-Whitney rank sum test. MeHg, THg, MeHg/THg ratio, and *hgcA* measurements are presented as means ± standard deviations. The MeHg/THg ratio was calculated by dividing THg by MeHg concentrations and multiplying by 100%.

NMDS analysis visualized differences in microbial community composition, with OTUs normalized using the Hellinger transformation (Legendre and Gallagher, 2001) and a Bray-Curtis dissimilarity matrix. NMDS plots were generated using the vegan package in R (Oksanen et al., 2019).

Diversity indices (species richness, Shannon's H, and Pielou's evenness) were computed with the vegan package in R and visualized using boxplots, with significance assessed by Mann-Whitney U tests.

Bar plots showed the relative abundance of the top 30 taxa in sediments and periphyton, with less abundant taxa grouped as 'others' and unassigned OTUs grouped and labeled.

The proportion of *hgcA*+ taxa in each habitat was expressed as a percentage of total *hgcA* abundance per habitat.

Redundancy analysis (RDA) related environmental parameters to microbial community structure, using the vegan package on a Bray-Curtis dissimilarity matrix. Environmental parameters included MeHg, THg, MeHg/THg, OM%, C/N ratio, various metals and elements (such as Na, Mg, Al, K, Ca, Sc, Cr, Mn, Fe, Co, Ni, Cu, Zn, V, Se, Sr, As, Mo, Y, Ag, Cd, Sb, Ba, Eu, La, Ce, Pr, Nd), and additional water variables like temperature, pH, oxygen, DOC, CO_2_, CH_4_, Cl^-^, F^-^, NO_3_^-^, SO_4_^-^ and nutrients.

Linear regression models assessed relationships between MeHg, THg, or MeHg/THg ratio against C/N ratio or OM content, with significant models checked for normality and homoscedasticity before plotting.

**Supplementary Material S6.**

Our sediment samples (subsamples of sediment cores) have a higher β-diversity (between-sample diversity) than our periphyton bulk samples (**Figure 2**, upper panel; **Supplementary Figures S7** and **S9**): Sediment samples are more dispersed and show certain site and depth patterns.

Sediment WEM replicates cluster together and show a depth pattern where deeper sediments have microbial communities more distinct from other sites, likely due to limited connectivity with surface water or shallow sediments, fostering independent microbial species sorting and greater site-to-site differences (Liao et al., 2021). CA and RDC samples do not cluster apart, but RDC samples show a depth pattern in the opposite direction of the WEM depth pattern (**Supplementary Figure S7**), indicating that deeper sediments have more divergent microbial communities. This pattern is supported by diversity indices, showing a slight but significant decrease in diversity with increasing sediment depth at RDC (**Supplementary Figure S8**). Deeper sediment layers face specific selective pressures, such as reduced oxygen and nutrient availability, which can favor the proliferation of certain microbial taxa over others, leading to decreased diversity.

Unlike the dispersed distribution of sediments, periphyton samples (grown on macrophytes or submerged wood) cluster together, indicating greater similarity among microbial communities regardless of site (**Figure 2**, upper panel, **Supplementary Figure S9**). Only WEM samples, which originate from a distinct ecosystem — a wetland with stagnant water dominated by macrophytes — slightly differ from the other sites, which are riverine environments with fewer aquatic plants (**Supplementary Figure S9**).


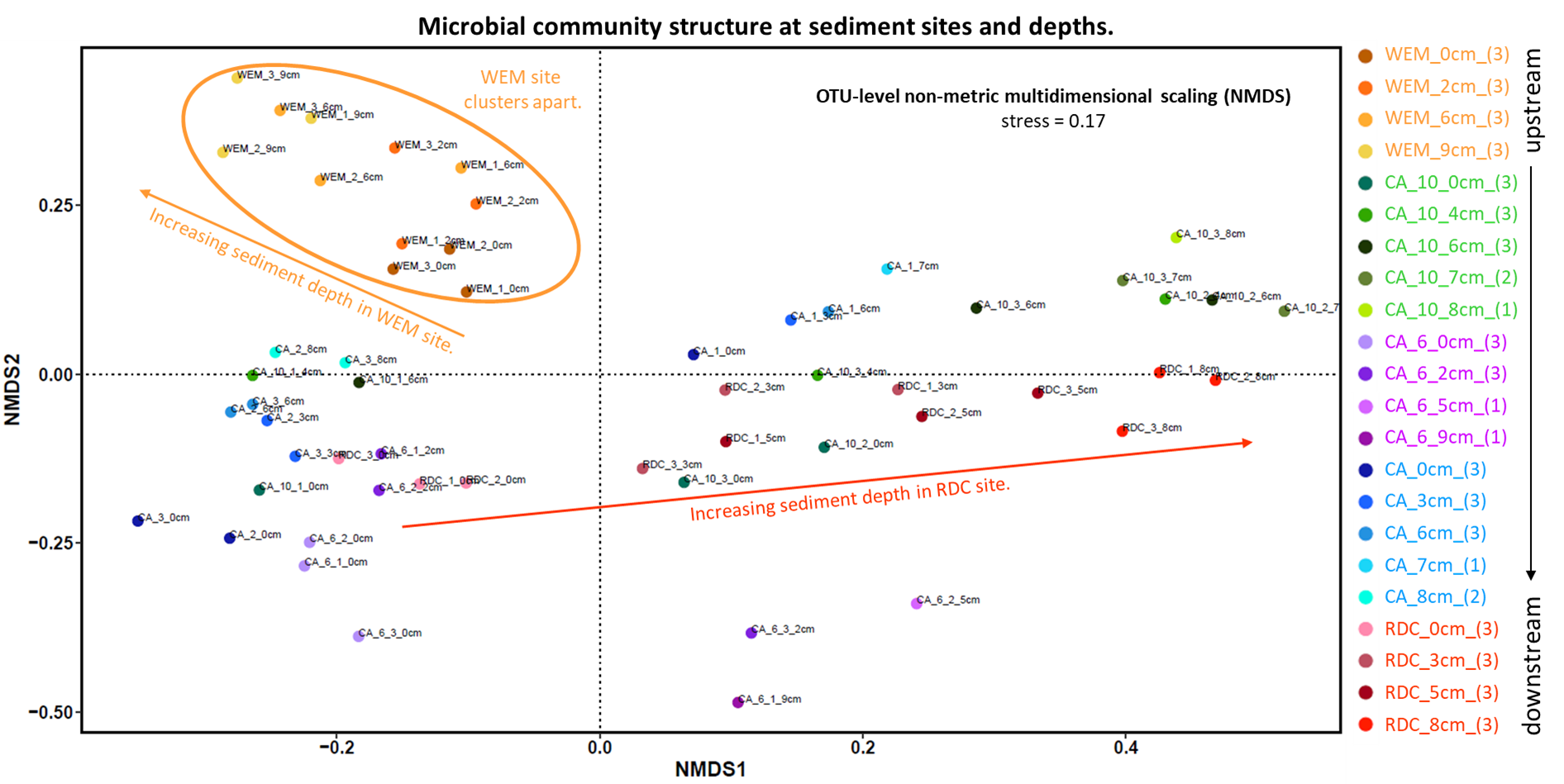


**Supplementary Figure S7.** Microbial community structure in sediments: OTU-level non-metric multidimensional scaling (NMDS) of all sediment samples. Sampling sites are color-coded as per the legend on the right side of the figure. Each dot represents 1-3 replicates (numbers indicated in brackets in the legend). The sampling depth is indicated in centimeters (cm).


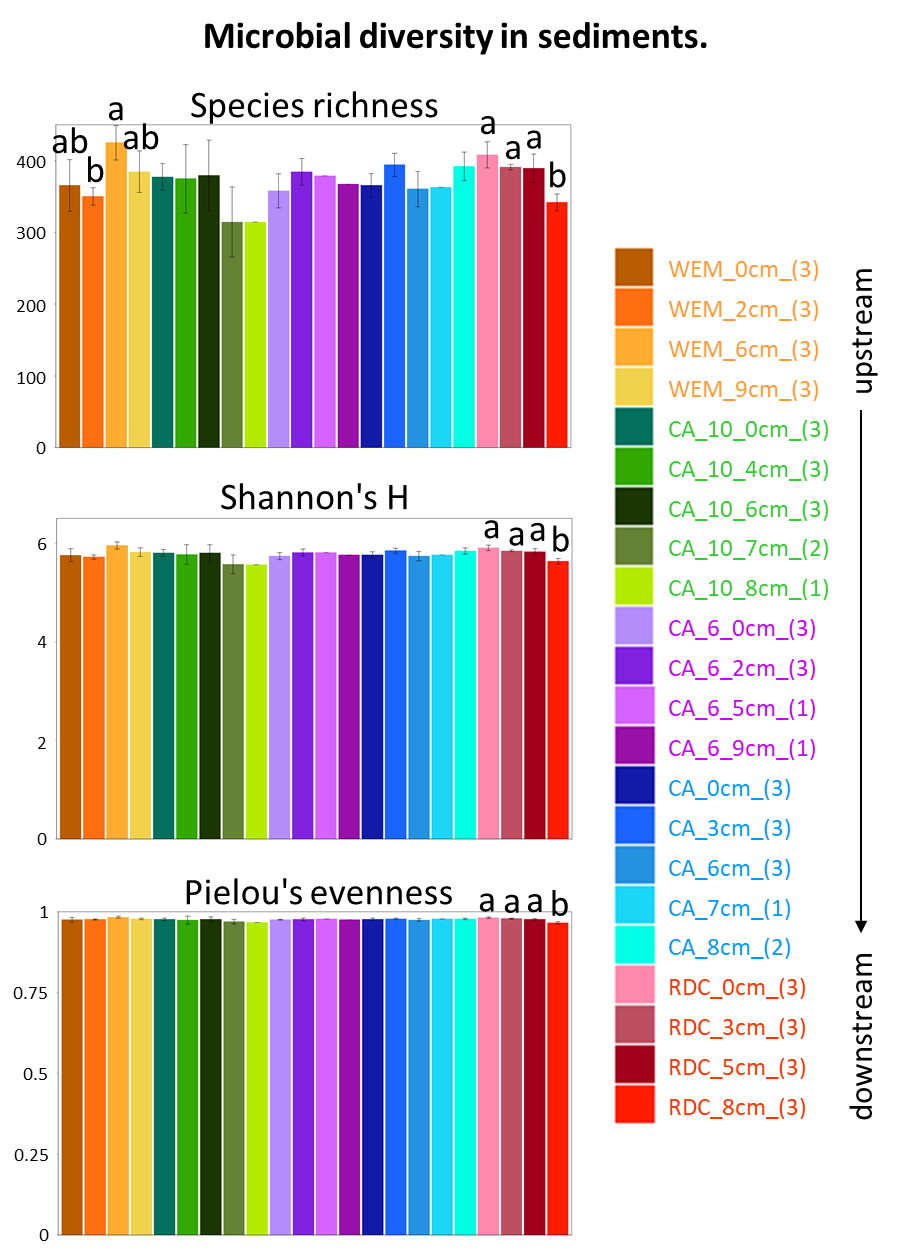


**Supplementary Figure S8.** Diversity indices, namely species richness, Shannon’s H, and Pielou’s evenness, for all sediment samples. Significant differences are indicated by letters. Samples are color-coded and ordered from upstream to downstream of the St-Maurice River. The number of replicates is indicated in brackets. Sediment depth is expressed in centimeters (cm).


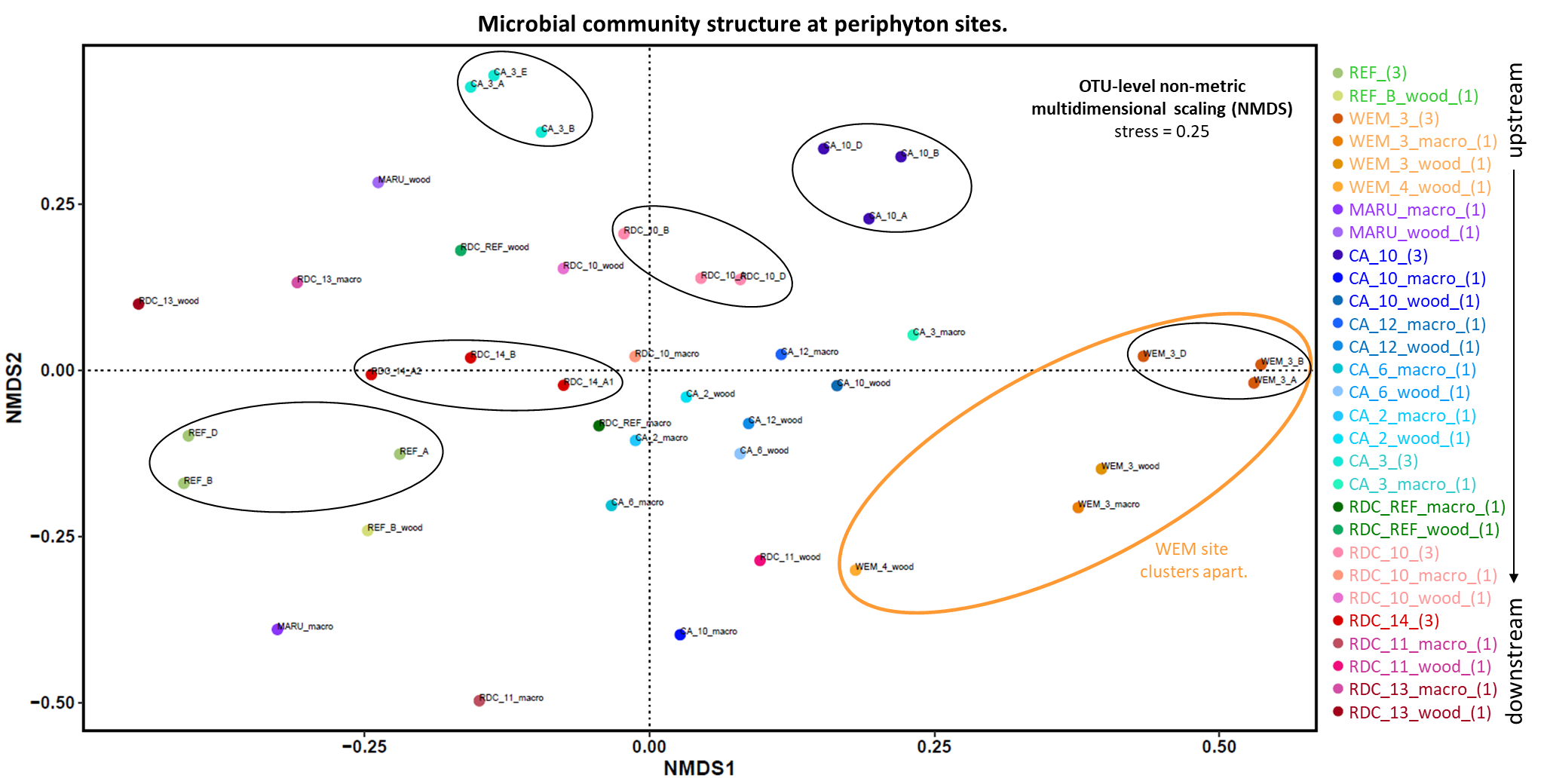


**Supplementary Figure S9.** Microbial community structure in periphyton: OTU-level non-metric multidimensional scaling (NMDS) of all periphyton samples. Sampling sites are color-coded as per the legend on the right side of the figure. Each dot represents 1-3 replicates (numbers indicated in brackets in the legend). Ellipses enclose replicate samples that exhibit similar visual groupings.

**Supplementary Material S10.**

Among the 15 most abundant phyla in sediments and periphyton, 12 phyla belong to bacteria (**Figure 3**). Proteobacteria are the most prevalent in both habitats. They are widely recognized to include species capable of methylating Hg (McDaniel et al., 2020). Other highly abundant bacterial phyla in both matrices are Chloroflexi, Bacteroidetes, Actinobacteria, Verrucomicrobia, Acidobacteria and Firmicutes_A, all known to include Hg methylators (McDaniel et al., 2020). One of the top 15 bacterial phyla unique to sediments is Desulfobacterota, identified as one of the most prevalent hosts of *hgcA* among Hg methylators in sediments in a prior study conducted within our project (Millera Ferriz et al., 2021).

Among the classes previously identified to host *hgcA* in the same sampling areas (Millera Ferriz et al., 2021; Leclerc et al., 2021), Bacteroidia and Anaerolineae rank among the top 15 most abundant classes in both sediments and periphyton in our study, suggesting that Hg methylators may be related in both, sediments and periphyton. In addition, Syntrophia and Methanomicrobia, previously identified as *hgcA* hosts in the same sampling areas, are among the most abundant in sediments but not in periphyton. This aligns with the higher *hgcA* abundance observed in sediments compared to periphyton (**Figure 1**).


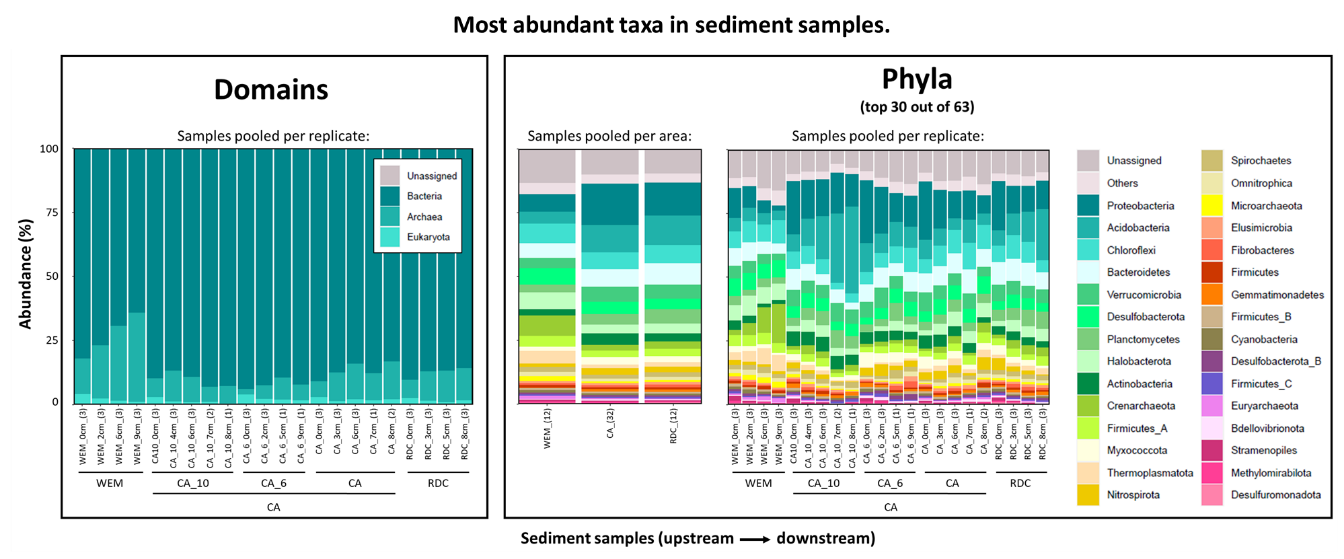


**Supplementary Figure S11.** Most abundant taxa in sediment samples at domain and phylum level. Taxa are color-coded as per the legend on the right side of the respective bar chart. Samples are pooled per replicate or per sampling area. Numbers in brackets indicate how many samples were pooled for each bar.


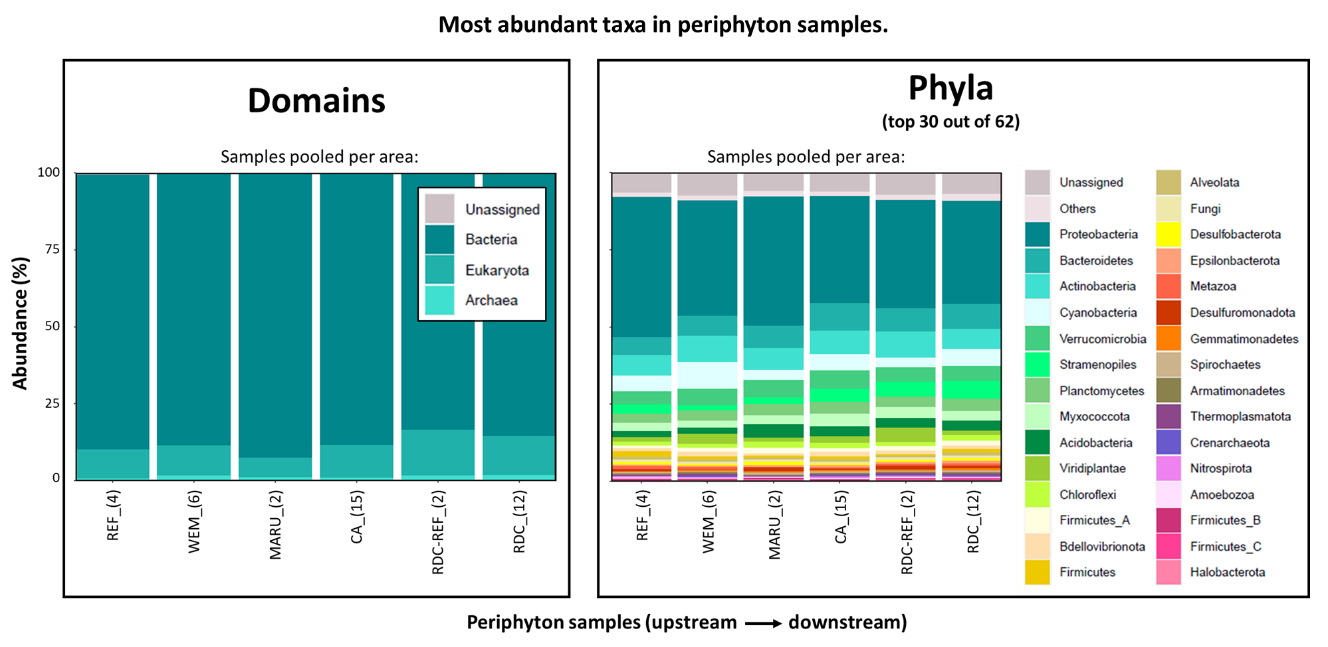


**Supplementary Figure S12.** Most abundant taxa in periphyton samples at domain and phylum level. Taxa are color-coded as per the legend on the right side of the respective bar chart. Samples are pooled per replicate or per sampling area. Numbers in brackets indicate how many samples were pooled for each bar.

**Supplementary Material S13.**

All *hgcA*+ methanogens are archaea and more prevalent in sediments (17% of sediment *hgcA*+ sequences) compared to periphyton (8%). This aligns with the higher abundance of archaea in sediments than in periphyton (**Figure 3**). Sulfate reducers exhibit similar relative abundances in sediments (25%) and periphyton (23%). Fermenters and iron reducers make up a larger proportion of the *hgcA*+ community in periphyton (35% and 12%) compared to sediments (26% and 1%). This may be due to the more even distribution of *hgcA*+ taxa in periphyton than in sediments (**Figure 4**), aligning with its higher microbial α-diversity (**Figure 2**, lower panel). However, oxygen levels are generally higher in periphyton than sediments, as periphyton produces and directly accesses oxygen from the water column (Stewart, 2012; Wetzel, 2001). This suggests that periphyton may be less favorable for fermenting and iron-reducing bacteria compared to sediments (Lovley, 1991; Schink, 1997). Thus, the *hgcA*+ community might not fully represent the composition of the overall microbial community in our study sites. Instead, Hg methylation seems to be a trait of specific, low-abundance taxa in the microbiome (Podar et al., 2015; Gionfriddo et al., 2020). *HgcA*+ classes with alternative metabolic pathways constitute only 4% in sediments and 9% in periphyton (**Figure 4**). Across all samples, *hgcA* was found in 38 taxonomic classes. Surprisingly, unassigned contigs make up 28% in sediment but only 14% in periphyton, despite periphyton being less studied for Hg methylators compared to sediments (Branfireun et al., 2020; Furutani and Rudd, 1980). The higher percentage of unassigned sequences in sediments cannot be attributed to the inclusion of multiple sediment depths, as no depth-related trends were observed in the proportion of unassigned sequences.

**Supplementary Material S14.**

Typically, significant environmental parameters or land use explain 10-20% of microbial community differences in published research, but this can vary based on the factors considered (Behera et al., 2019; Kraemer et al., 2020; Sauer et al. 2022). THg, MeHg, MeHg/THg, Fe, As and C/N show low but significant correlations to the sediment community structure (**Supplementary Figure S15**). Interestingly, all these parameters have been associated with Hg methylation in previous studies (Bravo et al., 2017; Feyte et al., 2012; Gionfriddo et al., 2023; Millera Ferriz et al., 2021).

Fe levels decrease with sediment depth (**Supplementary Figure S15**). The accumulation of Fe in the upper sediment layers is a common occurrence attributed to Fe(II) oxidation to Fe(III) oxides or hydroxides that precipitate upon exposure to oxygen at the anoxic-oxic sediment-water interface (Feyte et al., 2012; Tessier et al., 1996). The correlation between Fe and the microbial community structure may hence also indicate the potential impact of redox conditions on the microbial community, as commonly observed (Xu et al., 2021). This shift in redox conditions is also favorable to the methylation of Hg by anaerobic microbial communities which thrive at the anoxic-oxic interface (Si et al., 2022).


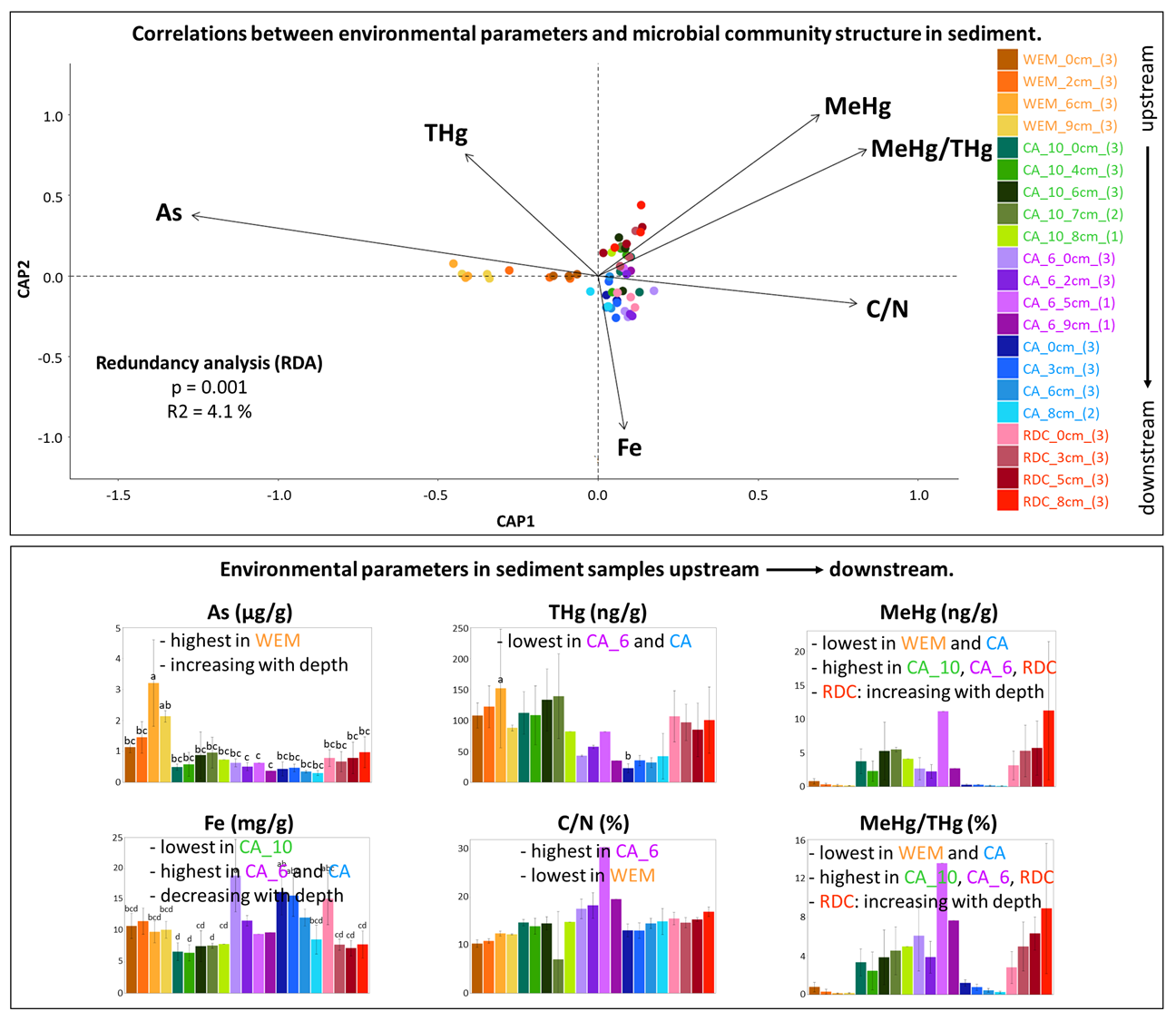


**Supplementary Figure S15.** The top panel presents a redundancy analysis (RDA) of sediment microbial community structure as a function of environmental parameters of sediments. Samples are color-coded according to the legend on the right. The number of replicates is given in brackets. Sediment depth is indicated in cm. The lower panel shows the sediment depth patterns for environmental parameters that affect the microbial community structure, as observed by the RDA. Significant differences in environmental parameters were assessed using ANOVA followed by a post-hoc Tukey's HSD test, and are indicated by letters.


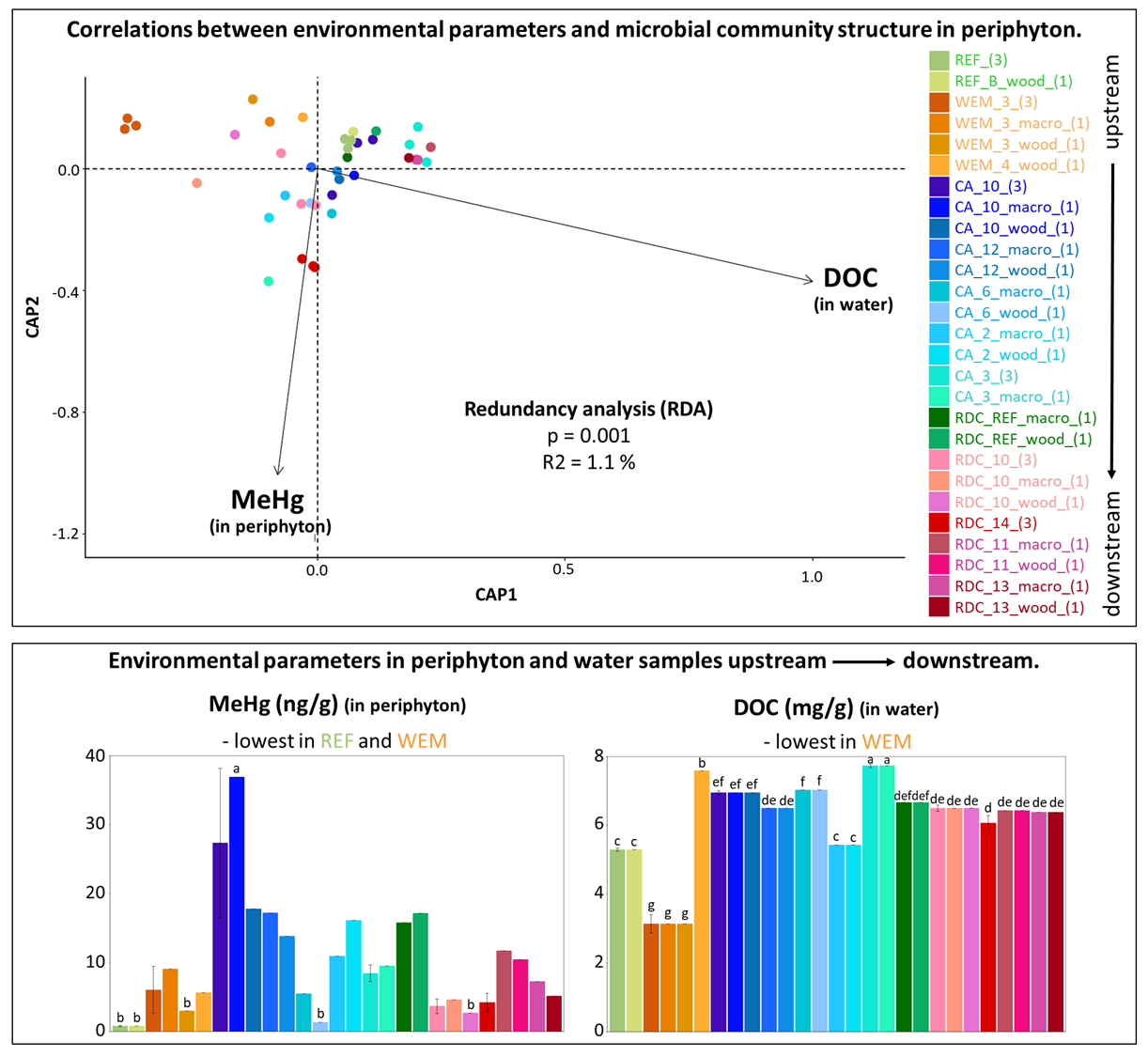


**Supplementary Figure S16.** The top panel presents a redundancy analysis (RDA) of periphyton microbial community structure as a function of environmental parameters of periphyton and water. Samples are color-coded according to the legend on the right. The number of replicates is given in brackets. The lower panel shows the patterns for environmental parameters that affect the microbial community structure, as observed by the RDA. Significant differences in environmental parameters were assessed using ANOVA followed by a post-hoc Tukey's HSD test, and are indicated by letters.

**Supplementary Material S17.**

Interestingly, the periphytic regression line has a steeper slope than that of sediment (**Figure 5**), likely due to the more easily degradable nature of aquatic OM in periphyton, as opposed to the more refractory terrigenous OM typically found in sediments (Bravo et al., 2017). In sediments, THg shows a positive correlation with OM%, whereas in periphyton, THg is not associated with OM content (**Figure 5**). This supports the observation that the sources of OM differ between sediments and periphyton: sediments are mainly influenced by the input of terrigenous OM and OM-bound THg from the watershed, whereas periphyton is predominantly composed of self-produced aquatic OM.


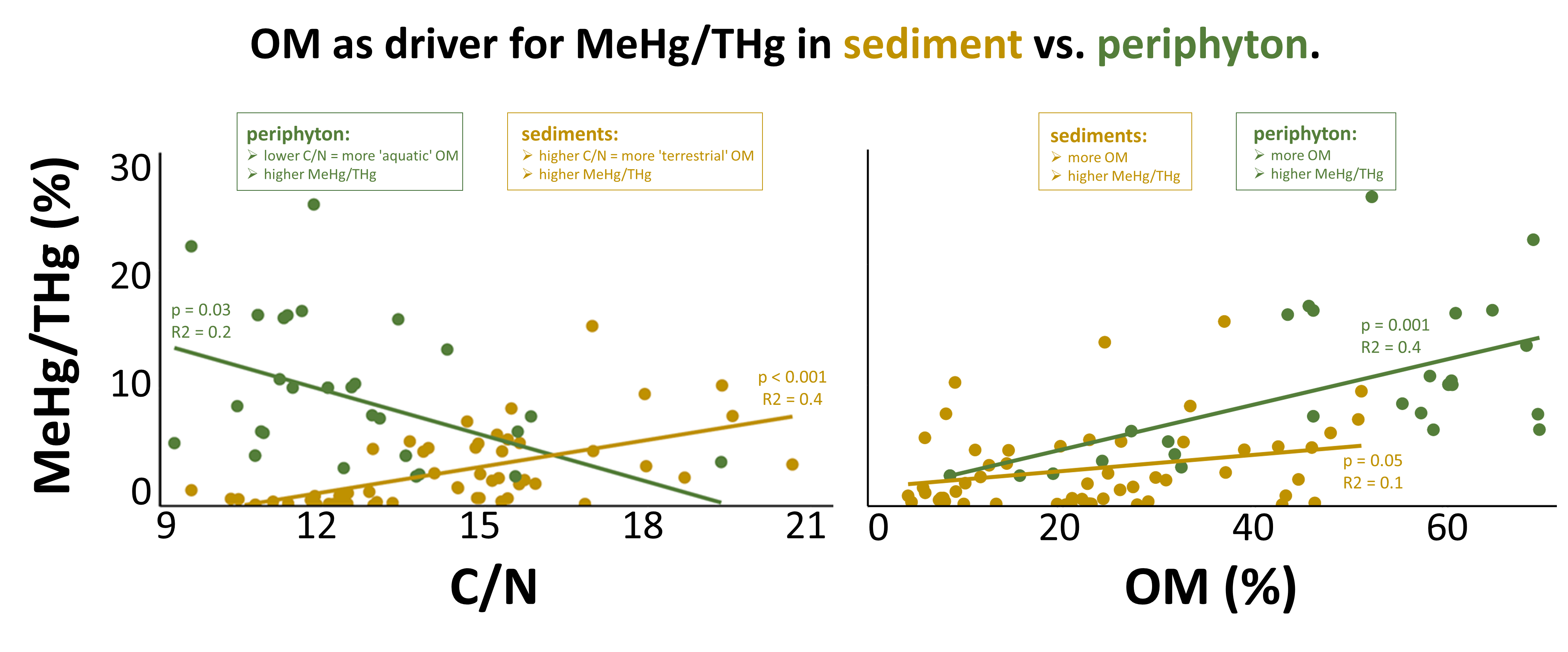


**Supplementary Figure S18.** OM quality (C/N) and quantity (OM %) as drivers for MeHg/THg in sediment (brown) and periphyton (green). Significant correlations among MeHg/THg, C/N, and OM are indicated by a regression line, accompanied by p and R2 values.

**Supplementary Figure S19.** Methane (CH_4_; µatmosphere; left side) and carbon dioxide concentrations (CO_2_; µatmosphere; right side) in water above sediments from CA (n = 17), WEM (n = 6), and RDC sites (n = 10). The dashed line represents dissolved CO_2_ concentrations expected at equilibrium with the atmosphere.

**Supplementary Material S20.**

Sediments harbor 55 ± 23 *hgcA*+ sequences per 1 million sequences, with a total of 23,710 *hgcA* sequences across all 56 sediment samples. In contrast, periphyton contains only 3.3 ± 4 *hgcA*+ sequences per 1 million sequences, with a total of 377 *hgcA* sequences across 19 out of 41 periphyton samples.

**
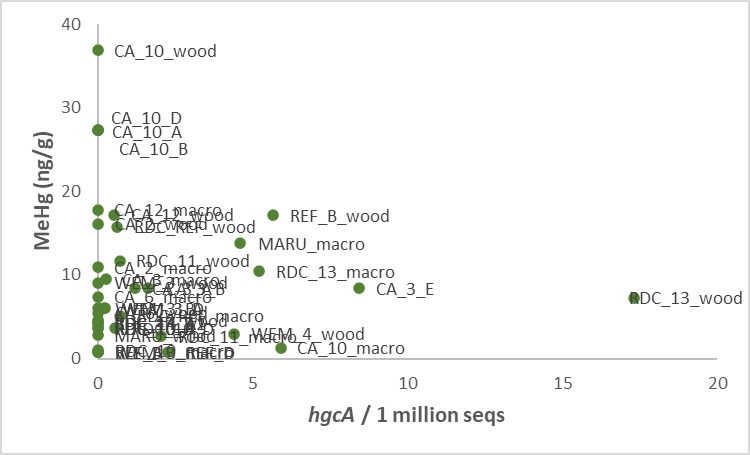
**

**Supplementary Figure S21.** Relationship between MeHg concentrations and *hgcA* abundance in periphyton.

**Supplementary Material S22.**

Hypoxic conditions were measured in the water column near the sediments in the CA area, with an average oxygen saturation of 37% over 24 hours (range 29-44%, n=47).

**Supplementary Table S23.** Percent oxygen saturation in CA and WEM areas measured using two autonomous YSI (EXO 2) multiparameter sonde installed horizontally; one approximately 30 cm below the water surface and one 30 cm above the sediments for 12 hours (CA) and 24 hours (WEM). The number of measurements over the submerged period is indicated (n).

| Site | Water column position | Min | Max | Mean | n | hours |
| --- | --- | --- | --- | --- | --- | --- |
| CA | Surface | 47 | 79 | 59 | 47 | 12 |
| CA | Deep | 29 | 44 | 37 | 47 | 12 |
| WEM | Surface | 64 | 91 | 76 | 92 | 24 |
| WEM | Deep | 64 | 90 | 75 | 92 | 24 |

**References**

Behera P, Mohapatra M, Kim JY, Adhya TK, Pattnaik AK, Rastogi G, 2019. Spatial and temporal heterogeneity in the structure and function of sediment bacterial communities of a tropical mangrove forest. Environmental Science and Pollution Research 26(4), 3893-3908, doi: 10.1007/s11356-018-3927-5.

Branfireun BA, Cosio C, Poulain AJ, Riise G, Bravo AG, 2020. Mercury cycling in freshwater systems - An updated conceptual model. Science of The Total Environment 745, 140906, doi: 10.1016/j.scitotenv.2020.140906.

Bravo A, Bouchet S, Tolu J, Björn E, Mateos-Rivera A, Bertilsson S, 2017. Molecular composition of organic matter controls methylmercury formation in boreal lakes. Nature Communications 8, 14255, doi: 10.1038/ncomms14255.

Downing JA, 1986. A regression technique for the estimation of epiphytic invertebrate populations. Freshwater Biology 16, 161-173, doi: 10.1111/j.1365-2427.1986.tb00961.x.

Feyte S, Gobeil C, Tessier A, Cossa D, 2012. Mercury dynamics in lake sediments. Geochimica et Cosmochimica Acta 82, 92-112, doi: 10.1016/j.gca.2011.02.007.

Furutani A, Rudd JW, 1980. Measurement of mercury methylation in lake water and sediment samples. Applied and Environmental Microbiology 40(4), 770-6, doi: 10.1128/aem.40.4.770-776.1980.

Gionfriddo CM, Soren AB, Wymore AM, Hartnett DS, Podar M, Parks JM, Elias DA, Gilmour CC, 2023. Transcriptional Control of *hgcAB* by an ArsR-Like Regulator in *Pseudodesulfovibrio* *mercurii* ND132. Applied and Environmental Microbiology 89(4), e0176822, doi: 10.1128/aem.01768-22.

Gionfriddo CM, Wymore AM, Jones DS, Wilpiszeski RL, Lynes MM, Christensen GA, Soren A, Gilmour CC, Podar M, Elias DA, 2020. An improved *hgcAB* primer set and direct high-throughput sequencing expand Hg-methylator diversity in nature. Frontiers in Microbiology 11, 541554, doi: 10.3389/fmicb.2020.541554.

Grasshoff K, Kremling K, Ehrhardt M, 2007. Methods of seawater analysis (3rd ed.). Wiley-VCH.

Kraemer SA, Barbosa da Costa N, Shapiro BJ, Fradette M, Huot Y, Walsh DA, 2020. A large-scale assessment of lakes reveals a pervasive signal of land use on bacterial communities. The ISME Journal 14(12), 3011-3023, doi: 10.1038/s41396-020-0733-0.

Leclerc M, Harrison MC, Storck V, Planas D, Amyot M, Walsh DA, 2021. Microbial diversity and mercury methylation activity in periphytic biofilms at a run-of-river hydroelectric dam and constructed wetlands. mSphere, 6(2), e00021-21, doi: 10.1128/mSphere.00021-21.

Legendre P, Gallagher ED, 2001. Ecologically meaningful transformations for ordination of species data. Oecologia 129(2), 271-280, doi: 10.1007/s004420100716.

Li D, Luo R, Liu C-M, Leung C-M, Ting H-F, Sadakane K, Yamashita H, Lam T-W, 2016. MEGAHIT v1. 0: a fast and scalable metagenome assembler driven by advanced methodologies and community practices. Methods 102, 3-11, doi: 10.1016/j.ymeth.2016.02.020.

Li H, Durbin R, 2010. Fast and accurate long-read alignment with burrows–wheeler transform. Bioinformatics 26(5), 589-595, doi: 10.1093/bioinformatics/btp698.

Liao W, Tong D, Li Z, Nie X, Liu Y, Ran F, Liao S, 2021. Characteristics of microbial community composition and its relationship with carbon, nitrogen and sulfur in sediments. Science of The Total Environment 795, 148848, doi: 10.1016/j.scitotenv.2021.148848.

Lovley DR, 1991. Dissimilatory Fe(III) and Mn(IV) reduction. Microbiological Reviews 55(2), 259-87, doi: 10.1128/mr.55.2.259-287.1991.

Markowitz VM, Ivanova NN, Szeto E, Palaniappan K, Chu K, Dalevi D, Chen I-MA, Grechkin Y, Dubchak I, Anderson I, Lykidis A, Mavromatis K, Hugenholtz P, Kyrpides NC, 2008. IMG/M: a data management and analysis system for metagenomes. Nucleic Acids Research 36(Suppl_1), D534-D538, doi: 10.1093/nar/gkm869.

McDaniel EA, Peterson BD, Stevens SLR, Tran PQ, Anantharaman K, McMahon KD, 2020. Expanded phylogenetic diversity and metabolic flexibility of mercury-methylating microorganisms. mSystems 5(4), e00299-20, doi: 10.1128/mSystems.00299-20.

Millera Ferriz L, Ponton DE, Storck V, Leclerc M, Bilodeau F, Walsh DA, Amyot M, 2021. Role of organic matter and microbial communities in mercury retention and methylation in sediments near run-of-river hydroelectric dams. Science of the Total Environment 774, 145686, doi: 10.1016/j.scitotenv.2021.145686.

Oksanen J, Blanchet FG, Friendly M, et al., 2022). Vegan: Community Ecology Package. R package version 2.6-4, https://github.com/vegandevs/vegan

Parks D, Chuvochina M, Waite D, Rinke C, Skarshewski A, Chaumeil P-A, Hugenholtz P, 2018. A standardized bacterial taxonomy based on genome phylogeny substantially revises the tree of life. Nature Biotechnology 36, 996-1004 (2018), doi: 10.1038/nbt.4229.

Podar M, Gilmour CC, Brandt CC, Soren A, Brown SD, Crable BR, Palumbo AV, Somenahally AC, Elias DA, 2015. Global prevalence and distribution of genes and microorganisms involved in mercury methylation. Science Advances 1, e1500675, doi: 10.1126/sciadv.1500675.

Ponton DE, Lavoie RA, Leclerc M, Bilodeau F, Planas D, Amyot M, 2021. Understanding food web mercury accumulation through trophic transfer and carbon processing along a river affected by recent run-of-river dams. Environmental Science and Technology 2, 55(5), doi: 10.1021/acs.est.0c07015.

Sauer HM, Hamilton TL, Anderson RE, Umbanhowar CE Jr, Heathcote AJ, 2022. Diversity and distribution of sediment bacteria across an ecological and trophic gradient. PLOS ONE 17(3), e0258079, doi: 10.1371/journal.pone.0258079.

Schink B, 1997. Energetics of syntrophic cooperation in methanogenic degradation. Microbiology and Molecular Biology Reviews 61(2), 262-80, doi: 10.1128/mmbr.61.2.262-280.1997.

Si L, Branfireun BA, Fierro J, 2022. Chemical oxidation and reduction pathways of mercury relevant to natural waters: A Review. Water 14(12), 1891, doi: 10.3390/w14121891.

St. Louis VL, Rudd JW, Kelly CA, Bodaly R, Paterson MJ, Beaty KG, Hesslein RH, Heyes A, Majewski AR, 2004. The rise and fall of mercury methylation in an experimental reservoir. Environmental Science and Technology 38, 1348-1358, doi: 10.1021/es034424f.

Stewart PM, 2012. Periphyton ecology: Exploiting cells on surfaces. In: Whitton, BA (Ed.), Ecology of Cyanobacteria II: Their Diversity in Space and Time. Springer, doi: 10.1007/978-94-007-3855-3_1.

Tessier A, Fortin D, Belzile N, DeVitre RR, Leppard GG, 1996. Metal sorption to diagenetic iron and manganese oxyhydroxides and associated organic matter: Narrowing the gap between field and laboratory measurements. Geochimica et Cosmochimica Acta 60(3), 387-404, doi: 10.1016/0016-7037(95)00413-0.

Wetzel RG, 2001. Limnology: Lake and River Ecosystems, 3rd Edition. Academic Press.

Woodcroft BJ, Aroney STN, Zhao R, Cunningham M, Mitchell JAM, Blackall L, Tyson GW, 2024. SingleM and Sandpiper: Robust microbial taxonomic profiles from metagenomic data (Version 0.16.0) [Computer software], doi: 10.1101/2024.01.30.578060.

Xu J, Liem-Nguyen V, Buck M, Bertilsson S, Björn E, Bravo AG, 2021. Mercury methylating microbial community structure in boreal wetlands explained by local physicochemical conditions. Frontiers in Environmental Science 8, doi: 10.3389/fenvs.2020.518662.
